# Optimized Colossal Near‐Field Thermal Radiation Enabled by Manipulating Coupled Plasmon Polariton Geometry

**DOI:** 10.1002/adma.202106097

**Published:** 2021-10-22

**Authors:** Kezhang Shi, Zhaoyang Chen, Xinan Xu, Julian Evans, Sailing He

**Affiliations:** ^1^ Centre for Optical and Electromagnetic Research State Key Laboratory of Modern Optical Instrumentation Zhejiang University Hangzhou 310058 China; ^2^ Centre for Optical and Electromagnetic Research ZJU‐SCNU Joint Center of Photonics South China Academy of Advanced Optoelectronics South China Normal University Guangzhou 510006 China; ^3^ Ningbo Research Institute Zhejiang University Ningbo 315100 China; ^4^ Department of Electromagnetic Engineering School of Electrical Engineering Royal Institute of Technology Stockholm S‐100 44 Sweden

**Keywords:** coupled plasmon polaritons, electric tuning, graphene Fermi level, multilayer graphene/SU8 heterostructures, nanoscale heat transfer manipulation

## Abstract

Collective optoelectronic phenomena such as plasmons and phonon polaritons can drive processes in many branches of nanoscale science. Classical physics predicts that a perfect thermal emitter operates at the black body limit. Numerous experiments have shown that surface phonon polaritons allow emission two orders of magnitude above the limit at a gap distance of ≈50 nm. This work shows that a supported multilayer graphene structure improves the state of the art by around one order of magnitude with a ≈1129‐fold‐enhancement at a gap distance of ≈55 nm. Coupled surface plasmon polaritons at mid‐ and far‐infrared frequencies allow for near‐unity photon tunneling across a broad swath of *k*‐space enabling the improved result. Electric tuning of the Fermi‐level allows for the detailed characterization and optimization of the colossal nanoscale heat transfer.

## Graphical abstract

Coupled plasmon polaritons excited by gap‐bridging multilayer graphene/SU8 heterostructures lead to near‐perfect photon‐tunneling probability and improve the state of the art by one order of magnitude in near‐field thermal radiation (NFTR). Electric tuning of the graphene Fermi‐level allows for optimized NFTR enhancement. Colossal energy transfer should inspire potential applications in thermophotovoltaic and so on.

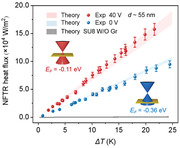

## 1 Introduction

Excitation of plasmons and phonon polaritons allow for colossal near‐field thermal radiation (NFTR) enhancement,^[^ ^1^, ^2^, ^3^, ^4^, ^5^, ^6^, ^7^ ^]^ topological polaritons,^[^ ^8^ ^]^ polaritonic manipulation,^[^ ^9^ ^]^ and other exotic nanoscale phenomena.^[^ ^10^, ^11^, ^12^, ^13^ ^]^ Over the last decades, numerous NFTR experiments^[^ ^14^, ^15^, ^16^, ^17^, ^18^, ^19^, ^20^ ^]^ have demonstrated that surface phonon polaritons allow emission two orders of magnitude above the black body (BB) limit at a gap distance of ≈50 nm. Compared with polar dielectric materials such as SiO_2_, graphene can excite surface plasmon polaritons (SPPs) in a broader infrared frequency region, providing an excellent channel for radiative heat transfer enhancement.^[^ ^1^, ^21^ ^]^ Careful control of graphene geometry can also enable extraordinary materials such as superconductors,^[^ ^22^ ^]^ correlated insulators,^[^ ^23^ ^]^ atomic‐scale ion transistors,^[^ ^24^ ^]^ ultrathin desalination membranes,^[^ ^25^ ^]^ and so on. Theoretically, one can further enhance the NFTR with a multilayer system^[^ ^26^, ^27^, ^28^ ^]^ via multiple surface‐states coupling such as multiple plasmons^[^ ^29^, ^30^ ^]^ or nonreciprocal graphene plasmons coupling.^[^ ^31^ ^]^ Here, preparing a gap‐bridging suspended crystal of multiple graphene sheets would allow for organized plasmon polariton modes. These coupled SPPs provide an extraordinary channel for NFTR enhancement since near‐perfect photon tunneling probability spans a large range of lateral wave‐vector. Graphene sheets have highly tunable coupled SPPs associated with the Fermi level in a linear Dirac band. Tuning the Fermi level can allow intersheet plasmon polaritons to support photon tunneling within desired mid‐ and far‐infrared frequency region, resulting in an optimized NFTR enhancement. However, preparing such a multilayer suspended system is challenging. Many support materials, such as SiO_2_, Si, or hBN, would confine those surface modes to a smaller lateral wave‐vector due to the higher index and loss of the structures. Here, we study a graphene/SU8/5‐layer heterostructure (Gr/SU8/5L) since SU8 is optically similar to vacuum within mid‐ and far‐infrared frequency region (Section S6, Supporting Information). Tuning the Fermi level allows for control of the shape of SPPs in *k*‐space, which allows for controlled NFTR enhancement. Due to the strong coupling of graphene SPPs, colossal enhancement of ≈1129‐fold compared to the BB limit at a gap distance of ≈55 nm was demonstrated between two Gr/SU8/5L heterostructures. The top relevant works we are aware of, show enhancement (vs their corresponding far‐field limits, which is smaller than the BB limit) at similar gap distances such as ≈100‐fold^[^ ^17^ ^]^ at ≈50 nm, ≈84‐fold^[^ ^18^ ^]^ at ≈42 nm, and ≈156‐fold^[^ ^19^ ^]^ at ≈50 nm. Hence, our Gr/SU8/5L heterostrucuture represents close to one order of magnitude improvement at similar gap distance. This giant heat transfer may inspire potential applications in thermophotovoltaic,^[^ ^32^ ^]^ thermal management,^[^ ^33^ ^]^ and novel communication systems.^[^ ^34^ ^]^

## 2 Theory

The net heat flux *Q* flowing from an emitter (with temperature *T*_1_) to a receiver (*T*_2_) at a gap distance *d* is determined by the integral of photon tunneling probabilities from fluctuational electrodynamics^[^ ^26^, ^27^, ^35^, ^36^, ^37^, ^38^ ^]^

(1) $$\begin{array}{l} Q\;\;=\;\;\frac{1}{4\pi^{2}}\int_{\infty }^{0}d\omega [\Theta (T_{1},\omega )\\ \;\;\;\;\;\;-\Theta (T_{2},\omega )]\;\;\cdot \;\;\left[\int_{k_{0}}^{0}\beta \sum_{j\;\;=\;\;s,p}\xi_{j}(\omega ,\beta ,d)d\beta +\int_{\infty }^{k_{0}}\beta \sum_{j\;\;=\;\;s,p}\xi_{j}(\omega ,\beta ,d)d\beta \right] \end{array}$$

where Θ(*T*,ω) = *ћω*/[exp(*ћω*/*k*_B_ *T*) − 1] is the mean energy of the Planck thermal harmonic oscillators and *k*_B_ is the Boltzmann constant. *ξ_j_*, as a function of angular frequency ω, lateral wave vector β and gap distance *d*, is the energy transmission coefficient between the emitter and receiver with value range from 0 to 1 for s‐ or p‐polarization modes

(2) $$\xi_{j}(\omega ,\beta )\;\;=\;\;\{\begin{array}{c} \frac{\left(1-|r_{j,e}{|}^{2})(1-|r_{j,r}{|}^{2}\right)}{|1-r_{j,e}r_{j,r}e^{2ik_{z0}d}{|}^{2}},\beta <k_{0}\\ \frac{4Im(r_{j,e})Im(r_{j,r})e^{2ik_{z0}d}}{|1-r_{j,e}r_{j,r}e^{2ik_{z0}d}{|}^{2}},\beta >k_{0} \end{array}$$

where *r_j,e_* and *r_j,r_* are the Fresnel reflection coefficients of the emitter and receiver, respectively, and *k*_z0_ is the *z*‐component of the wave vector in vacuum (*k*_0_). When β > *k*_0_, ξ_p_ represents the photon tunneling probability of p‐polarization modes. Maximizing the available area of *k*‐space is the primary way to pursue colossal NFTR.

For a finite‐thickness structure with single layer, the basic reflection coefficients are^[^ ^26^ ^]^

(3) $$\begin{array}{c} r_{s}\;\;=\;\;\frac{r_{s01}+r_{s12}(1+r_{s01}+r_{s10})e^{2ik_{z}^{\left(1s\right)}t_{1}}}{1-r_{s10}r_{s12}e^{2ik_{z}^{\left(1s\right)}t_{1}}}\\ r_{p}\;\;=\;\;\frac{r_{p01}+r_{p12}(1-r_{p01}-r_{p10})e^{2ik_{z}^{\left(1p\right)}t_{1}}}{1-r_{p10}r_{p12}e^{2ik_{z}^{\left(1p\right)}t_{1}}} \end{array}$$

where *t*_1_ is the thickness of layer one, and $r_{s(p),n_{1},n_{2}}$ is the Fresnel reflection coefficient at the interface between layer *n*_1_ and layer *n*_2_ for s‐ or p‐polarization modes. When the number of layers increases, e.g., the Gr/SU8/5L heterostructures on the SiO_2_/Si substrate, reflection coefficients should be an iteration of Equation (3). For infinite‐layer structures, reflection coefficients could be found from ref. ^[^ ^26^ ^]^.

Graphene was treated as surface current with conductivity σ described as^[^ ^26^, ^39^, ^40^ ^]^

(4) $$\sigma \;\;=\;\;\frac{2ie^{2}k_{B}T\ln[2\cosh[E_{F}/(2k_{B}T)]]}{(\omega +i/\tau )\pi \hbar^{2}}\;\;+\;\;\frac{e^{2}}{4\hbar }\left[G\left(\frac{\hbar \omega }{2}\right)+\frac{i4\hbar \omega }{\pi }I\right]$$

where *G*(δ) = sinh (δ/*k*_B_ *T*)/[cosh (*E*_F_/*k*_B_ *T*) + cosh (δ/*k*_B_ *T*)]and $I\;\;=\;\;\overset{0}{\underset{\infty }{\int \;}}\left[G(\delta )-G\left(\frac{\hbar \omega }{2}\right)\right]/[{\left(\hbar \omega \right)}^{2}-4\delta^{2}]d\delta$. *E*_F_ is the graphene Fermi level (also known as chemical potential). *e* is the elementary charge, *ћ* = *h*/2π is the reduced Planck constant. τ = 100 fs chosen in all of our calculations is a collision time,^[^ ^1^, ^7^, ^41^, ^42^ ^]^ which is a typical value associated with carrier‐carrier intraband collisions and phonon emission^[^ ^42^ ^]^ and has been used in previous experimental work with similar temperature.^[^ ^1^, ^7^ ^]^ σ is an even function of *E*_F_, i.e., σ (*E*_F_) = σ (−*E*_F_).

The dielectric function of SU8 was retrieved from our reflectance spectra experiments (see Section S6, Supporting Information) and modeled as multiple Lorentz–Drude oscillators^[^ ^43^ ^]^

(5) $$\varepsilon_{SU8}(wn)\;\;=\;\;\varepsilon_{\inf}+\;\;\sum_{m}^{x\;\;=\;\;1}\;\;\frac{A_{1x}}{1-{\left(\frac{wn}{A_{2x}}\right)}^{\;\;2}-i\left(\frac{wn}{A_{2x}}\right)A_{3x}}$$

where fitting parameters *A*_1x_, *A*_2x_, and *A*_3x_ are the strength, resonance frequency, and damping factor of the *x*th Lorentz–Drude oscillator, respectively. ε_inf_ is the value of ε_SU8_ with infinite wave number (*wn*). *m* = 14 was used in this work. Dielectric functions of SiO_2_ and Si were taken from ref. ^[^ ^44^ ^]^.

## 3 Results and Discussion

**Figure 1:**
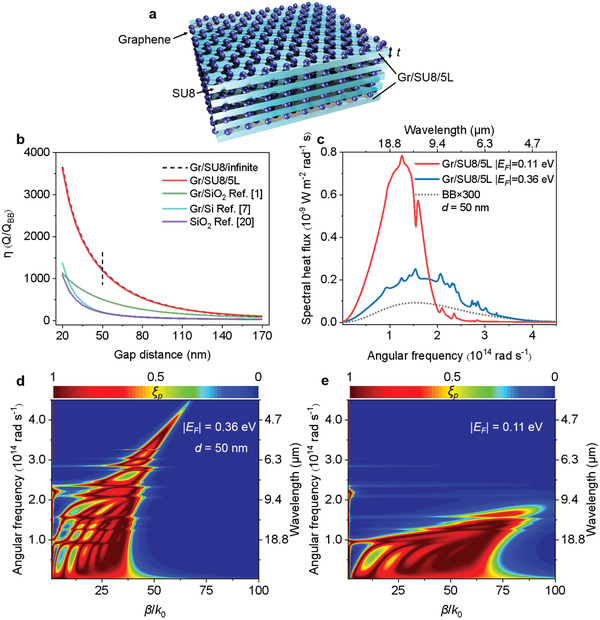
Schematic illustration, calculation, and analysis of NFTR enhancement. a) Schematic illustration of the heterostructure consisting of five layers of graphene with SU8 spacers (Gr/SU8/5L). The thickness *t* of each SU8 spacer is 90 nm. b) Heat flux *Q* as a function of gap distance from 20 to 170 nm (normalized with the corresponding BB limit) for Gr/SU8/infinite, Gr/SU8/5L, Gr/SiO_2_ (from ref. ^[^ ^1^ ^]^), Gr/Si (from ref. ^[^ ^7^ ^]^), and SiO_2_ (from ref. ^[^ ^20^ ^]^). Graphene Fermi levels |*E*_F_| were set to 0.11 eV in the calculation. c) Spectral heat flux for Gr/SU8/5L heterostructures at *d* = 50 nm with different |*E*_F_|. The spectrum corresponding to BB exchange (determined by Planck's Law and multiplied by 300) was illustrated. Temperatures *T*_1_ = 313.15 K and *T*_2_ = 303.15 K were used for calculation in (b) and (c). d,e) Contour maps of ξ_p_ for two Gr/SU8/5L heterostructures at *d* = 50 nm, with |*E*_F_| = 0.36 eV for (d) and |*E*_F_| = 0.11 eV for (e).

The Gr/SU8/5L consists of five layers of graphene‐covered SU8 structure with thickness *t* = 90 nm for each SU8 layer (**Figure** **1**a). The enhancement factor η (heat flux *Q* normalized with the corresponding BB limit) between two identical Gr/SU8/5L heterostructures (with graphene Fermi level |*E*_F_| = 0.11 eV) within a gap distance range from 20 to 170 nm was displayed in Figure 1b. SiO_2_ was considered as a substrate for the Gr/SU8/5L heterostructures. For comparison, η of Gr/SU8/infinite heterostructures (with periodic graphene‐covered SU8), graphene‐covered SiO_2_ (from ref. ^[^ ^1^ ^]^), graphene‐covered Si (from ref. ^[^ ^7^ ^]^), SiO_2_ (from ref. ^[^ ^20^ ^]^) were also calculated. η of Gr/SU8/5L heterostructures is quite close to that of the infinite layers limit and remarkably better than that of all other structures within this gap distance range. When *d* = 50 nm, η of Gr/SU8/5L heterostructures reaches 1188.7, which is 2.4‐fold, 6.3‐fold, and 6.6‐fold of that of Gr/SiO_2_, Gr/Si, and SiO_2_ structures, respectively. The enhancement of the Gr/SU8/5L heterostructures dominates for all near‐field distances. The optimal Fermi level and layer thickness depend on the gap distance. For example at 20 nm, η = 5160, with *t* = 33 nm and |*E*_F_| = 0.08 eV, or at 7 nm, η = 1.83 × 10^4^, with *t* = 13 nm and |*E*_F_| = 0.05 eV, which is still larger than other polar materials such as SiO_2_ (η ≈ 0.9× 10^4^), indicating the robustness of the multilayer system at smaller gap distances. Hence, the optimal thickness of each layer scales with the gap distance which highlights the importance of the periodic organization of the plasmon modes. The optimal thickness of the SU8 layer is 55 nm at the 50 nm vacuum gap, where η could reach ≈1265. Due to the difficulty of sample fabrication, we choose SU8 spacers with a thickness of 90 nm.

The influence of the graphene Fermi level can be seen from the computed spectral heat flux between two identical Gr/SU8/5L heterostructures (Figure 1c). The spectrum corresponding to BB exchange (multiplied by 300) was also calculated for comparison. When |*E*_F_| = 0.36 eV (which corresponds to the experiment with 0 V bias voltage), the spectrum covers a broader frequency range but has smaller value with η = 679.5. When |*E*_F_| = 0.11 eV, the spectrum is redshifted with much larger spectral heat flux in the mid‐ and far‐infrared frequency range, showing that the tunability of graphene Fermi level allows for spectral regulation and overall optimization. Contour maps of ξ_p_ (*β *> *k*_0_) with corresponding |*E*_F_| at *d* = 50 nm (Figure 1d,e) show that coupled SPPs in multiple graphene sheets with |*E*_F_| = 0.36 and 0.11 eV can both support almost continuous near‐unity ξ_p_, allowing for efficient energy transfer from the emitter to the receiver. Near‐unity ξ_p_ enabled by coupled graphene SPPs from the interacting graphene sheets is restricted to branches, which split at a lower β and merge at a larger β in a broad frequency range, demonstrating the significant role of coupled SPPs in enhancing near‐field heat transfer. ξ_p_ is not “smooth” since the imaginary part of the SU8 dielectric function shows small peaks within an angular frequency range from 1.0 × 10^14^ rad s^−1^ to 3.5 × 10^14^ rad s^−1^. However, this has little effect on the NFTR heat flux, which is an integration over all angular frequencies and lateral wave vectors. Using multiple sheets allows for many branches which can fill the available *k*‐space. For |*E*_F_| = 0.36 eV, these coupling modes with relatively large β enable near‐unity ξ_p_ covering a quite broad frequency range (from 2 × 10^12^ to 4 × 10^14^ rad s^−1^). While when |*E*_F_| = 0.11 eV, more coupled SPPs with larger β (up to 100 *k*_0_) are supported. Continuous near‐unity ξ_p_ covers the spectral region where most of the thermal photons are radiated near room temperature,^[^ ^45^ ^]^ showing the better performance. These strongly coupled SPPs enable high photon state density near the surface of the structures and are primarily responsible for radiation spectra in Figure 1c.

**Figure 2:**
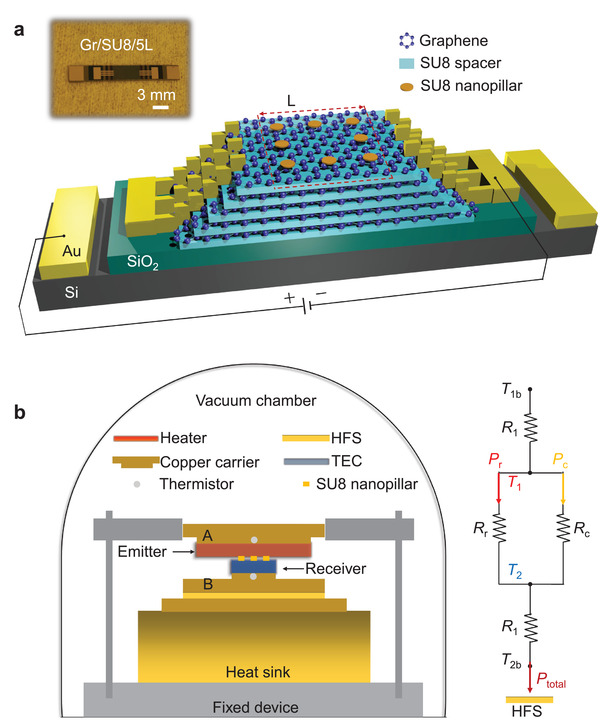
Schematic illustrations of our samples and NFTR experimental setup. a) Photo and schematic diagram of the Gr/SU8/5L sample. The Red‐dashed square (with side length *L* = 3 mm) is the active area of the receiver. Au on the Si substrate was used as the positive electrode while Au on the SiO_2_ film was the negative electrode. The applied voltage was adjusted to control the graphene Fermi level. b) Side‐view schematic illustration of the NFTR experimental setup in a vacuum chamber. The equivalent thermal circuit was also displayed. *T*_1b_ and *T*_2b_ were backside temperatures of the emitter and receiver, measured by two thermistors embedded in the copper carriers A and B, respectively. *T*_1_ and *T*_2_ were the top surface temperatures of the emitter and receiver estimated by the measured sum thermal resistance (*R*_1_) of the sample, thermal conductive adhesive, and copper carrier (Section S3, Supporting Information). *T*_2_ was maintained at 303.15 ± 0.5 K with 0 and 5 V bias voltages, or at 304.15 ± 0.5 K with 20 and 40 V bias voltages. *R*_r_ and *R*_c_ are the equivalent NFTR resistance and thermal conduction resistance, respectively.

Gr/SU8/5L heterostructures were prepared on a ≈ 300 nm thick SiO_2_ layer (on 525 µm thick Si substrate) as the emitter and receiver. As illustrated in **Figure** **2**a, SU8 spacers and graphene sheets were stacked layer by layer with graphene contacted with Au/Ti electrodes to control the Fermi levels^[^ ^46^, ^47^, ^48^ ^]^ (see Section S2, Supporting Information). SU8 nanopillars with identical thickness within a range from 58 to 63 nm and diameter of 20 µm were fabricated on the receiver at the center of the active area (≈ 3 × 3 mm^2^, red‐dashed square). The experimental setup consists of an emitter and receiver with fixed gap distance separated by SU8 nanopillars as shown in Figure 2b. The equivalent thermal circuit was illustrated. *P*_total_ = *P*_r_ + *P*_c_ is the total heat power directly measured by the heat flux sensor (HFS). *P*_c_ = *S κ* |Δ*T*|/*d* computed based on Fourier's Law is the heat conduction contributed by eight SU8 nanopillars. *S* is the sum area of eight SU8 nanopillars, κ is the thermal conductivity^[^ ^3^ ^]^ (0.2 W m^−1^ K^−1^), and *d* is the gap distance as well as the thickness of SU8 nanopillars. This thermal conductivity of the SU8 nanopillars has been experimentally verified with a pair of SiO_2_/Si samples (with a gap distance of ≈ 81.3 nm) and the average value was found to be 0.207 ± 0.01 W m^−1^ K^−1^ (see Section S5, Supporting Information). One may also perform this measurement with other low emissivity materials.^[^ ^49^ ^]^ Hence, measured radiation heat flux of the samples (with active area Λ) could be obtained by

(6) $$Q_{\exp}\;\;=\;\;\frac{\left(P_{total}-P_{c}\right)}{\Lambda }$$

*P*_r_ represents the NFTR power which could be simply converted to experimental heat flux *Q*_exp_ by dividing by the active area of the emitter and receiver.

**Figure 3:**
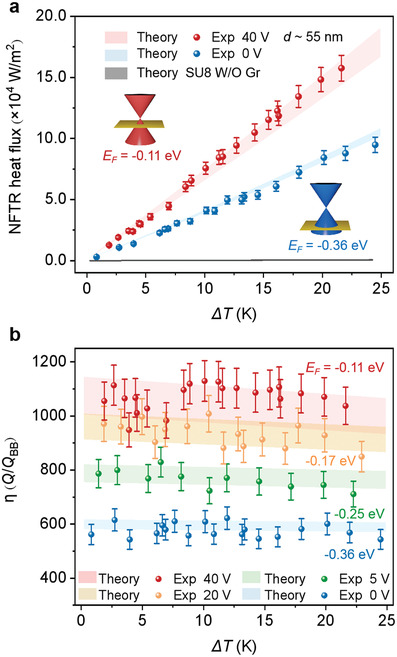
Measured and calculated NFTR heat flux with different Δ*T* at different graphene Fermi levels. a) Measured NFTR heat flux of our Gr/SU8/5L heterostructures as Δ*T* varies at *E*_F_ = −0.11 eV (40 V bias voltage) and *E*_F_ = −0.36 eV (0 V bias voltage) within a gap distance range from ≈ 52.4 to ≈ 57 nm (i.e., *d* ≈ 55 nm, see Section S5, Supporting Information). This gap distance range accounts for the range of the theoretical calculation of the NFTR. The gray band shows the theoretical calculation of the NFTR between a pair of 450 nm thick SU8 film (on SiO_2_/Si) without graphene sheets. The inset shows the schematic illustration of two different graphene Fermi levels (in different colors) due to different hole doping. b) Enhancement (normalized with the corresponding BB limit) at different *E*_F_ with different Δ*T*. Colored bands in (a) and (b) show the corresponding theoretical predictions at *T*_2_ = 304.15 K for the cases of *E*_F_ = −0.11 eV and *E*_F_ = −0.17 eV, and *T*_2_ = 303.15 K for the cases of *E*_F_ = −0.25 eV and *E*_F_ = −0.36 eV. Error bars were plotted due to the uncertainty of the active area (from 2.9 × 2.9 to 3.1 × 3.1 mm^2^). Both the emitter and receiver were controlled at the same bias voltages by external sources.

*E*_F_ was a fitting parameter which was found to be −0.36 eV at 0 V bias voltage (indicating a hole doping of graphene) which was similar to the reference value determined by the Raman spectra of another identical Gr/SU8/5L sample (Section S2, Supporting Information). With bias voltage up to 40 V, more electrons flowed into the graphene sheets from the negative electrode, leading to a reduced hole doping level, where *E*_F_ reaches −0.11 eV. The gap distance *d* was determined by the thickness and mechanical properties of the SU8 nanopillars and was found to be within a range from ≈ 52.4 to ≈ 57 nm (i.e., *d* ≈ 55 nm, Section S5, Supporting Information). The NFTR heat flux between two Gr/SU8/5L heterostructures with temperature difference (Δ*T*) varying from ≈ 1 to ≈ 24.5 K with bias voltage 0 and 40 V show excellent agreement with the theoretical predictions (**Figure** **3**a). Remarkable enhancement was observed at *E*_F_ = −0.11 eV, as coupled SPPs enable almost continuous near‐unity ξ_p_ with larger β in *k*‐space for the mid‐ and far‐infrared frequency region. For comparison, the NFTR between a pair of 450 nm thick SU8 film (on SiO_2_/Si) without graphene sheets was also calculated (gray band in Figure 3a). The small radiative heat flux of the SU8 film indicates that the colossal near‐field thermal radiation is attributed to the graphene plasmonic enhancement. Bias voltages of 5 and 20 V were also applied for the heat flux measurement, corresponding to a fitted *E*_F_ of −0.25 and −0.17 eV, respectively. Figure 3b shows the enhancement factor η as a function of temperature gradient at different bias voltages, which demonstrates the potential for continuous tuning. At a gap distance of ≈ 55 nm, the heat flux of the Gr/SU8/5L heterostructures with *E*_F_ = −0.11 eV could exceed the BB limit by three orders of magnitude. For example, at *ΔT* ≈ 10 K, η of the Gr/SU8/5L heterostructures with *E*_F_ = −0.11 eV is 1129, which is ≈ 1.85‐fold above the same conditions with *E*_F_ = −0.36 eV. In our case, the contribution of the heat conduction *P*_c_ (vs the total heat power *P*_total_) is relatively small, only ≈ 13% at *E*_F_ = −0.11 eV and ≈ 20% at *E*_F_ = −0.36 eV, respectively (see Section S7, Supporting Information). The results of the NFTR power *P*_r_ (obtained by subtracting the calculated heat conduction *P*_c_, whose thermal conductivity has also been verified by our experiment in a simple case, from the measured total heat power *P*_total_) should be fairly close to the actual NFTR power *P*_r_.

## 4 Conclusion

In conclusion, we have experimentally demonstrated a three orders of magnitude enhancement compared to the BB limit with a pair of graphene‐covered SU8 five layers heterostructures at a gap distance of ≈ 55 nm. Due to the continuous near‐unity photon tunneling probability enabled by coupled graphene SPPs, the enhancement is around one order of magnitude better than SiO_2_ or SiC at a similar gap. Tuning the Fermi level shows the ability to manipulate the distribution of near‐unity photon tunneling to be across a broad frequency band or broad range of *k*‐vector. The results demonstrate that the Gr/SU8/5L heterostructures gives the strongest known experimental NFTR for a gap distance in the regime of tens of nanometers. These exciting results should inspire further exploration of graphene‐based near‐field thermal radiation devices, technological development, novel materials, and structures design.

## 5 Experimental Section

### Experimental Setup

The NFTR experimental setup was sealed in a vacuum chamber with all heat flux measurements performed in a high vacuum environment at a pressure of ≈ 2.8 × 10^−5^ Pa. As shown in Figure 2b, a miniature ceramic heater embedded in copper carrier A was used to control the adjustable temperature *T*_1_ for the emitter, while the heat through the receiver (carried by copper carrier B) was brought to the heat sink by an embedded temperature electric controller (TEC, 1‐12705, Realplay), maintaining a temperature *T*_2_ for the receiver. Two platinum resistance temperature detectors (thermistor, M222 class 1/3B, Heraeus, Germany) embedded beneath the top surface of the copper carriers were used to directly obtain the backside temperatures (*T*_1b_ and *T*_2b_) of the emitter and receiver, so that *T*_2_ and *T*_1_ could be estimated by a relationship: *T*_2_ (*T*_1_) = *T*_2b_ (*T*_1b_) ± *P*_total_ *R*_1_ (Section S3, Supporting Information). A 20 × 20 × 0.4 mm^3^ ultrathin HFS (HS‐20, Captec, France) was embedded below copper carrier B for the heat flux measurement with heat flux values displayed by an external heat flux meter (HFM‐8, Captec, France). A total 95 g mass (including copper carrier A) was added onto the device to strengthen the contact and mechanical stability of the system^[^ ^3^, ^7^ ^]^ (not shown), resulting in a small compression of the gap distance (i.e., the SU8 nanopillars) with ≈5.6 to ≈6 nm (see Section S5, Supporting Information). Two posts fixed on an optical breadboard were employed to improve the mechanical stability and alignment between the emitter and receiver. The whole experimental setup including the vacuum chamber was placed on an optical platform in a clean room.

### Sample Fabrication and Characterization

The emitter and receiver are fabricated using a standard UV lithography technique and wetting transfer method (for graphene sheets). The surface curvature and surface residue of the sample are characterized by a laser interferometer (ZYGO OMP‐035/M) and atom force microscope. The thickness of the SU8 spacers is measured by a film thickness measuring instrument (Filmetrics, F40‐UV) (see the Supporting Information for details).

## Conflict of Interest

The authors declare no conflict of interest.

## Author Contributions

K.S. and Z.C. contributed equally to this work. S.H. conceived and supervised the work. K.S. conceived the work and performed the calculations. K.S. and Z.C. built up the measurement system and performed the experiments. K.S., Z.C., and X.X. fabricated the emitter and receiver devices. K.S., Z.C., and X.X. performed the sample characterization. The paper was discussed and written by K.S., J.E., and S.H. with comments and input from all authors.

## Supporting information

Supporting Information

## Data Availability

The data that support the findings of this study are available from the corresponding author upon reasonable request.

## References

[1] K. Z. Shi , Y. C. Sun , Z. Y. Chen , N. He , F. L. Bao , J. Evans , S. L. He , Nano Lett. 2019, 19, 8082. 31646871 10.1021/acs.nanolett.9b03269

[2] L. X. Zhu , A. Fiorino , D. Thompson , R. Mittapally , E. Meyhofer , P. Reddy , Nature 2019, 566, 239. 30760913 10.1038/s41586-019-0918-8

[3] J. DeSutter , L. Tang , M. Francoeur , Nat. Nanotechnol. 2019, 14, 751. 31263192 10.1038/s41565-019-0483-1

[4] W. Du , J. Yang , S. Zhang , N. Iqbal , Y. D. Dang , J. B. Xu , Y. G. Ma , Nano Energy 2020, 78, 105264.

[5] M. Ghashami , H. Y. Geng , T. Kim , N. Iacopino , S. K. Cho , K. Park , Phys. Rev. Lett. 2018, 120, 175901. 29756825 10.1103/PhysRevLett.120.175901

[6] M. Lim , J. Song , S. S. Lee , B. J. Lee , Nat. Commun. 2018, 9, 4302. 30327494 10.1038/s41467-018-06795-w PMC6191454

[7] J. Yang , W. Du , Y. S. Su , Y. Fu , S. X. Gong , S. L. He , Y. G. Ma , Nat. Commun. 2018, 9, 4033. 30279411 10.1038/s41467-018-06163-8 PMC6168489

[8] G. W. Hu , Q. D. Ou , G. Y. Si , Y. J. Wu , J. Wu , Z. G. Dai , A. Krasnok , Y. Mazor , Q. Zhang , Q. L. Bao , C. W. Qiu , A. Alù , Nature 2020, 582, 209. 32528096 10.1038/s41586-020-2359-9

[9] Q. Zhang , Q. D. Ou , G. W. Hu , J. Y. Liu , Z. G. Dai , M. S. Fuhrer , Q. L. Bao , C. W. Qiu , Nano Lett. 2021, 21, 3112. 33764791 10.1021/acs.nanolett.1c00281

[10] J. Lee , D. J. Jeon , J. S. Yeo , Adv. Mater. 2021, 202006606, 10.1002/adma.202006606. 33891781

[11] Q. S. Guo , F. Guinea , B. C. Deng , I. Sarpkaya , C. Li , C. Chen , X. Ling , J. Kong , F. N. Xia , Adv. Mater. 2017, 29, 1700566. 10.1002/adma.201700566 28621022

[12] M. G. Gong , M. Alamri , D. Ewing , S. M. Sadeghi , J. Z. Wu , Adv. Mater. 2020, 32, 2002163. 10.1002/adma.202002163 32449564

[13] Y. W. Zhu , S. Murali , W. W. Cai , X. S. Li , J. W. Suk , J. R. Potts , R. S. Ruoff , Adv. Mater. 2010, 22, 3906. 20706983 10.1002/adma.201001068

[14] E. Rousseau , A. Siria , G. Jourdan , S. Volz , F. Comin , J. Chevrier , J. J. Greffet , Nat. Photonics 2009, 3, 514.

[15] S. Shen , A. Narayanaswamy , G. Chen , Nano Lett. 2009, 9, 2909. 19719110 10.1021/nl901208v

[16] K. Kim , B. Song , V. Fernandez‐Hurtado , W. Lee , W. H. Jeong , L. J. Cui , D. Thompson , J. Feist , M. T. H. Reid , F. J. Garcia‐Vidal , J. C. Cuevas , E. Meyhofer , P. Reddy , Nature 2015, 528, 387. 26641312 10.1038/nature16070

[17] B. Song , D. Thompson , A. Fiorino , Y. Ganjeh , P. Reddy , E. Meyhofer , Nat. Nanotechnol. 2016, 11, 509. 26950244 10.1038/nnano.2016.17

[18] R. St‐Gelais , L. Zhu , S. Fan , M. Lipson , Nat. Nanotechnol. 2016, 11, 515. 26950243 10.1038/nnano.2016.20

[19] A. Fiorino , D. Thompson , L. X. Zhu , B. Song , P. Reddy , E. Meyhofer , Nano Lett. 2018, 18, 3711. 29701988 10.1021/acs.nanolett.8b00846

[20] H. Salihoglu , W. Nam , L. Traverso , M. Segovia , P. K. Venuthurumilli , W. Liu , Y. Wei , W. J. Li , X. F. Xu , Nano Lett. 2020, 20, 6091. 32628493 10.1021/acs.nanolett.0c02137

[21] O. Ilic , M. Jablan , J. D. Joannopoulos , I. Celanovic , H. Buljan , M. Soljacic , Phys. Rev. B 2012, 85, 155422.

[22] Y. Cao , V. Fatemi , S. Fang , K. Watanabe , T. Taniguchi , E. Kaxiras , P. Jarillo‐Herrero , Nature 2018, 556, 43. 29512651 10.1038/nature26160

[23] Y. Cao , V. Fatemi , A. Demir , S. Fang , S. L. Tomarken , J. Y. Luo , J. D. Sanchez‐Yamagishi , K. Watanabe , T. Taniguchi , E. Kaxiras , R. C. Ashoori , P. Jarillo‐Herrero , Nature 2018, 556, 80. 29512654 10.1038/nature26154

[24] Y. H. Xue , Y. Xia , S. Yang , Y. Alsaid , K. Y. Fong , Y. Wang , X. Zhang , Science 2021, 372, 501. 33926952 10.1126/science.abb5144

[25] P. Sun , K. Wang , H. Zhu , Adv. Mater. 2016, 28, 2287. 26797529 10.1002/adma.201502595

[26] K. Z. Shi , F. L. Bao , S. L. He , ACS Photonics 2017, 4, 971.

[27] B. Zhao , B. Guizal , Z. M. Zhang , S. H. Fan , M. Antezza , Phys. Rev. B 2017, 95, 245437.

[28] Y. Zhang , H. L. Yi , H. P. Tan , ACS Photonics 2018, 5, 3739.

[29] H. Iizuka , S. H. Fan , Phys. Rev. Lett. 2018, 120, 063901. 29481235 10.1103/PhysRevLett.120.063901

[30] Y. Zhang , C. H. Wang , H. L. Yi , H. P. Tan , J. Quant. Spectrosc. Radiat. Transfer 2018, 221, 138.

[31] C. L. Zhou , L. Qu , Y. Zhang , H. L. Yi , Phys. Rev. B 2020, 102, 245421.

[32] G. R. Bhatt , B. Zhao , S. Roberts , I. Datta , A. Mohanty , T. Lin , J. M. Hartmann , R. St‐Gelais , S. H. Fan , M. Lipson , Nat. Commun. 2020, 11, 2545. 32439917 10.1038/s41467-020-16197-6 PMC7242323

[33] D. Thompson , L. Zhu , E. Meyhofer , P. Reddy , Nat. Nanotechnol. 2020, 15, 99. 31873289 10.1038/s41565-019-0595-7

[34] S. A. Biehs , R. Messina , P. S. Venkataram , A. W. Rodriguez , J. C. Cuevas , P. Ben‐Abdallah , Rev. Mod. Phys. 2021, 93, 025009.

[35] K. Z. Shi , R. Liao , G. J. Cao , F. L. Bao , S. L. He , Opt. Express 2018, 26, A591. 29801276 10.1364/OE.26.00A591

[36] Z. M. Zhang , Nano/Microscale Heat Transfer, McGraw‐Hill, New York 2007.

[37] M. P. Bernardi , D. Milovich , M. Francoeur , Nat. Commun. 2016, 7, 12900. 27682992 10.1038/ncomms12900 PMC5056409

[38] X. H. Wu , C. J. Fu , Z. M. Zhang , J. Heat Transfer 2020, 142, 072802.

[39] R. Messina , P. Ben‐Abdallah , Sci. Rep. 2013, 3, 1383. 23474891 10.1038/srep01383 PMC3593225

[40] L. Falkovsky , S. Pershoguba , Phys. Rev. B 2007, 76, 153410.

[41] K. Z. Shi , F. L. Bao , N. He , S. L. He , Int. J. Heat Mass Transfer 2019, 134, 1119.

[42] F. Bonaccorso , Z. Sun , T. Hasan , A. Ferrari , Nat. Photonics 2010, 4, 611.

[43] H. W. Verleur , J Opt. Soc. Am. 1968, 58, 1356.

[44] E. D. Palik , Handbook of Optical Constants of Solids, Academic Press, San Diego, CA 1985.

[45] V. Fernández‐Hurtado , F. J. García‐Vidal , S. Fan , J. C. Cuevas , Phy. Rev. Lett. 2017, 118, 203901. 10.1103/PhysRevLett.118.203901 28581797

[46] Z. Fei , A. S. Rodin , G. O. Andreev , W. Bao , A. S. McLeod , M. Wagner , L. M. Zhang , Z. Zhao , M. Thiemens , G. Dominguez , M. M. Fogler , A. H. Castro Neto , C. N. Lau , F. Keilmann , D. N. Basov , Nature 2012, 487, 82. 22722866 10.1038/nature11253

[47] S. Pisana , M. Lazzeri , C. Casiraghi , K. S. Novoselov , A. K. Geim , A. C. Ferrari , F. Mauri , Nat. Mater. 2007, 6, 198. 17293849 10.1038/nmat1846

[48] N. H. Thomas , M. C. Sherrott , J. Broulliet , H. A. Atwater , A. J. Minnich , Nano Lett. 2019, 19, 3898. 31141664 10.1021/acs.nanolett.9b01086

[49] N. M. Ravindra , B. Sopori , O. H. Gokce , S. X. Cheng , A. Shenoy , L. Jin , S. Abedrabbo , W. Chen , Y. Zhang , Int. J. Thermophys. 2001, 22, 1593.

