# Supplementary material for: Optimized Colossal Near‐Field Thermal Radiation Enabled by Manipulating Coupled Plasmon Polariton Geometry
Source: Adv Mater. 2021 Oct 22;33(52):2106097. doi: 10.1002/adma.202106097 (PMC11468567; doi:10.1002/adma.202106097)
Supplement: Supplementary file 1 — Supporting Information [file ADMA-33-2106097-s001.pdf]

ADVANCED MATERIALS

Supporting Information

for *Adv. Mater.,* DOI: 10.1002/adma.202106097

Optimized Colossal Near-Field Thermal Radiation Enabled by Manipulating Coupled Plasmon Polariton Geometry

*Kezhang Shi, Zhaoyang Chen, Xinan Xu, Julian Evans, and Sailing He**

Supporting Information

Optimized colossal near-field thermal radiation enabled by manipulating coupled plasmon polariton geometry

*Kezhang Shi, Zhaoyang Chen, Xinan Xu, Julian Evans and Sailing He**

Dr. K. Shi, X. Xu, Dr. J. Evans, Prof. S. He
Centre for Optical and Electromagnetic Research
State Key Laboratory of Modern Optical Instrumentation
Zhejiang University
Hangzhou 310058, China
E-mail: sailing@kth.se

Z. Chen
Centre for Optical and Electromagnetic Research
ZJU-SCNU Joint Center of Photonics
South China Academy of Advanced Optoelectronics
South China Normal University
Guangzhou 510006, China

Prof. S. He
Ningbo Research Institute
Zhejiang University
Ningbo 315100, China

Prof. S. He
Department of Electromagnetic Engineering
School of Electrical Engineering
Royal Institute of Technology
Stockholm S-100 44, Sweden

Keywords: coupled plasmon polaritons; nanoscale heat transfer manipulation; graphene Fermi level; electric tuning; multilayer graphene/SU8 heterostructures;

TABLE OF CONTENTS

Supporting Information Section 1: **Sample fabrication**

Supporting Information Section 2: **Sample characterization**

Supporting Information Section 3: **Temperature calibration**

Supporting Information Section 4: **Experimental system and measurement**

Supporting Information Section 5: **Gap distance and SU8 thermal conductivity**

Supporting Information Section 6: **Dielectric function of SU8 spacer**

Supporting Information Section 7: **Effect of heat conduction of SU8 nano pillars**

Supporting Information Section 8: **Effect of number of layers of Gr/SU8 heterostructures for NFTR**

1. Sample fabrication

(a) For receiver samples

Samples are fabricated using a standard UV lithography technique and wetting transfer method (for graphene sheets) as schematically illustrated in Figure S1. **Step 1**: A clean ~ 300-nm-thick SiO_2 film on a Si wafer was dealt with Hexamethyl Disilazane (HMDS) for 20 min to obtain a hydrophobic surface which promotes photoresist adhesion. **Step 2**: A photoresist (AZ5214, Resemi) was spin coated onto the wafer surface at 500 rpm for 6 s followed by 2000 rpm for 50 s and then baked for 5 min at 90°C, serving as protective layer with thickness of ~ 2 μm. **Step 3**: UV lithography produced nine 25 × 25 mm^2 blocks containing square patterns with size of 16 × 16 mm^2 which were then immersed in a developing solution (300MIF) and cleaned several times with deionized water (DW) **Step 4**: The wafer was dried using a nitrogen gas gun and cut into nine identical samples. **Step 5**: Inductively coupled plasma (ICP) thoroughly etched the SiO_2 except for the region protected by the photoresist (with 16 × 16 mm^2 area). **Step 6**: To clean the photoresist, the samples were ultrasonicated in NMP solution for 1 h, followed by isopropyl alcohol (IPA) for 10 min and DW for 10 min, and soaked in Piranha solution (volume ratio, concentrated sulfuric acid (H_2SO_4) : hydrogen peroxide (H_2O_2) = 7 : 3) to eliminate organic residues, and rinsed several times with DW. **Step 7**: The samples were spin coated with dilute SU8-3005 photoresist (mass ratio, SU8 : solvent cyclopentanone = 1 : 13) at 500 rpm for 6 s followed by 3300 rpm for 50 s, and then baked for 30 s at 95°C. **Step 8**: 10 × 10 mm^2 SU8 spacers (marked as the fifth SU8 layer) with thickness of ~ 90 nm were fabricated after the UV lithography with an exposure dose of 250 mJ and post baked at 95 °C twice for each with 30 s, followed by immersion in a developing solution (PGMEA) and standard cleaning procedures. **Step 9**: A sheet of graphene (on copper foil) covered by a paraffin protective layer with a rectangular pattern (with size of L_1 > 10 mm and L_2 ≥ 10 mm for the fifth graphene layer) was transferred to the sample using a wetting transfer method[1] (WTM, see details in **Step 9.1** to **Step 9.9** below). **Step 10**: The Paraffin protective layer was then removed by soaking several times in trichloroethylene (C_2HCl_3). The samples were then baked in an oven at 95°C for 3 h (optional procedure to melt large paraffin particles and enhance the contact between graphene and substrate) and soaked in C_2HCl_3 again, followed by very careful cleaning with IPA and DW to avoid graphene breakage. **Step 11**: For better spin coating of the fourth SU8 layer, the samples were baked at 95 °C for 25 min to reduce the moisture on the surface. The same parameters were employed as in Step 7 except that the samples were dried naturally for 30 min after spin coating instead of baking for 30 s at 95°C. **Step 12:** The UV lithography procedures from Step 8 were

repeated, and the 9 × 9 mm^2 SU8 spacers were well-prepared for transferring the fourth layer of graphene (with size of L_1 > 10 mm and L_2 = 9 mm). **Steps 13-17:** Fabrication of the third (shown in Figure S1), second, and first layer of both SU8 spacers and graphene sheets, with procedures repeated from Steps 9-12. The areas of SU8 spacers for the third, second, and first layer were 8 × 8 mm^2, 7 × 7 mm^2, and 6 × 6 mm^2. Hence, lengths of graphene sheet in Step 9 were correspondingly change to L_2 = 8 mm, 7 mm, and 6 mm, respectively. **Step 18**: To prepare the Au electrodes, AZ5214 photoresist was spin coated on the samples at 500 rpm for 6 s followed by 2000 rpm for 50 s and then baked for 2 min at 90 °C. **Step 19**: Patterns were obtained after a standard UV lithography and very careful cleaning. **Step 20**: A 5-nm-thick titanium adhesion layer was deposited on the samples, followed by the deposition of a 95-nm-thick Au film, using an E-beam evaporation method. **Step 21**: Metal films were removed using a lift-off method in acetone solution. **Step 22**: To prepare the SU8 nano pillars. Dilute SU8-3005 (mass ratio, SU8 : solvent cyclopentanone = 1 : 21) was spin coated on the samples at 500 rpm for 6 s followed by 3000 rpm for 50 s, leading to a homogeneous thickness of ~ 58 nm to ~ 63 nm. The same procedures as in step 8 were used to obtain eight SU8 nano pillars with diameter of 20 μm. Emitter samples were fabricated with identical methods without the SU8 nano pillars.

Samples close to the center of the SiO_2/Si wafer were chosen as the emitter and receiver unless the complicated fabrication procedures produced unusable units. SU8 spacers, Au electrodes, SU8 nano pillars were aligned centrally with alignment marks within an estimated deviation of ~ 2 μm, which was negligible for millimeter-scale plates. [**Caution:** Piranha solution is highly corrosive and explosive. Trichloroethylene (C_2HCl_3) is volatile poisonous organic vapour. Make sure these solutions are used inside a fume cupboard and wear a gas mask]

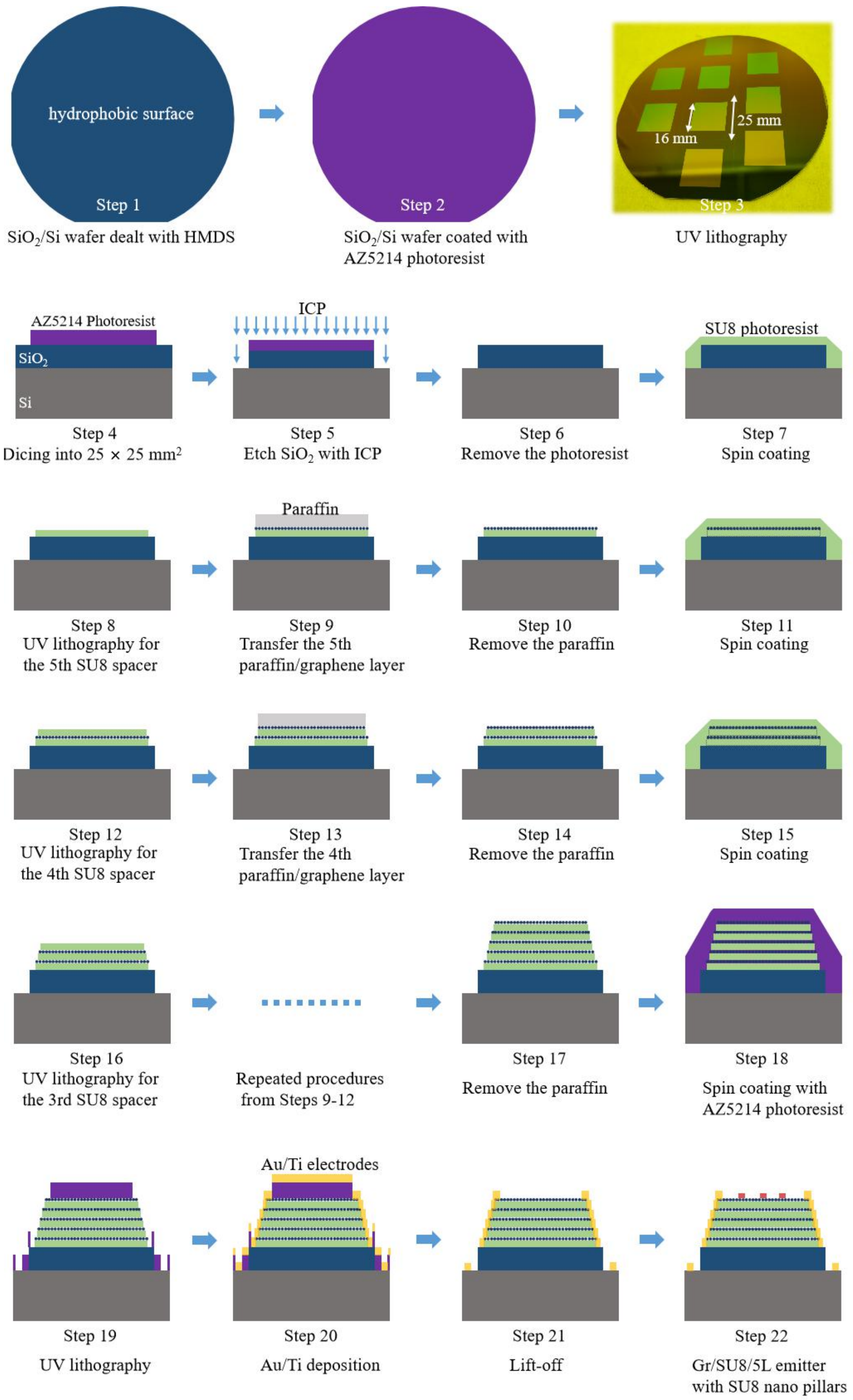
hydrophobic surface
Step 1
SiO2/Si wafer dealt with HMDS
Step 2
SiO2/Si wafer coated with AZ5214 photoresist
25 mm
16 mm
Step 3
UV lithography
AZ5214 Photoresist
SiO2
Si
Step 4
Dicing into 25 × 25 mm2
ICP
Step 5
Etch SiO2 with ICP
Step 6
Remove the photoresist
SU8 photoresist
Step 7
Spin coating
Step 8
UV lithography for the 5th SU8 spacer
Paraffin
Step 9
Transfer the 5th paraffin/graphene layer
Step 10
Remove the paraffin
Step 11
Spin coating
Step 12
UV lithography for the 4th SU8 spacer
Step 13
Transfer the 4th paraffin/graphene layer
Step 14
Remove the paraffin
Step 15
Spin coating
Step 16
UV lithography for the 3rd SU8 spacer
Repeated procedures from Steps 9-12
Step 17
Remove the paraffin
Step 18
Spin coating with AZ5214 photoresist
Step 19
UV lithography
Au/Ti electrodes
Step 20
Au/Ti deposition
Step 21
Lift-off
Step 22
Gr/SU8/5L emitter with SU8 nano pillars

Figure S1. Schematic illustration of fabrication of the Gr/SU8/5L sample. Figure in Step 3 is a photo of the SiO_2/Si wafer with patterns of AZ5214 photoresist after UV lithography. Step 4 to step 22 show the side views of the sample with size of 25 × 25 mm^2. The schematic illustrations are not drawn to scale.

(b) Wetting transfer method for graphene sheets: **Steps 9.1-9.9**

Based on Ref. [1] and our experimental conditions, paraffin (Sigma-Aldrich, 18634) was chosen as a protective layer when transferring graphene sheets. **Step 9.1**: Graphene on copper foil (CVD commercial product, graphene/Cu/backside graphene) was cut down and put onto a quartz or Si substrate and carefully fixed by adhesive tape on the edges. **Step 9.2**: After heating in an oven for 60 min at 80 °C, paraffin liquid was dropped onto the graphene sample on the substrate and solidified. **Step 9.3**: Paraffin was spin coated onto the graphene after melting by a heat gun.[1] **Step 9.4**: Graphene coated by paraffin film was cut into the required size. The copper foil was etched by 1 (mol L^{-1}) $FeCl_3$ solution for 8 min and the back side of the copper foil was washed with DW to remove extra graphene (on the back side of the copper foil). **Step 9.5**: The paraffin/graphene/Cu sample was then transferred to a new $FeCl_3$ solution for 70 min to sufficiently etch the copper foil. **Step 9.6**: A clean hydrophilic substrate (e.g., a SiO_2/Si substrate) was employed to transfer the floating paraffin/graphene into clean DW twice and allowed to sit for 10 min for each time to remove the $FeCl_3$ residues. **Step 9.7**: To obtain a relatively flat graphene sheet,[1] the floating paraffin/graphene was transferred to a new clean DW and heated in a water bath at ~ 35°C to 38 °C for more than 1 h. **Step 9.8**: Paraffin/graphene was finally transferred onto the target sample very carefully. To avoid tearing of the fragile paraffin/graphene (at ~ 35 °C to 38 °C), this operation was done after the DW naturally cooled down to ~ 25 °C. **Step 9.9**: The sample was then placed in an oven at 40 °C for 24 h to remove the residual water.

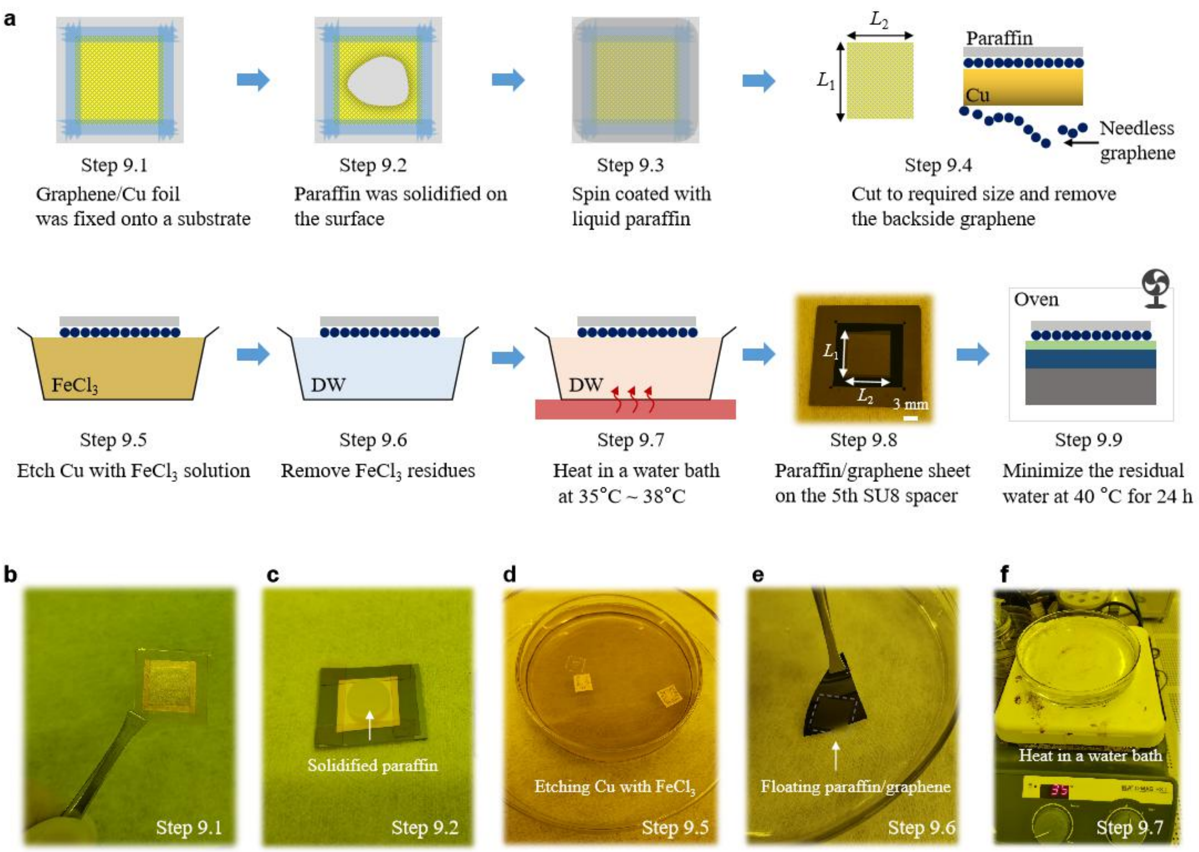

Figure S2. Schematic illustration and photos of transferring a graphene sheet to a SU8 spacer. a) Step 9.1 to Step 9.9 show the details of the wetting transfer method for graphene sheet. b-f) Photos of Step 9.1, Step 9.2, Step 9.5, Step 9.6, and Step 9.7, respectively. The schematic illustrations in Figure S2a are not drawn to scale.

(c) Wires for bias voltage device.

The sample after Step 22 was cut to a required size (~ 25 × 3 mm^2). As depicted in Figure S3, two wires (schematic diagram) were attached to the sample as positive (connected to Au electrode on Si substrate) and negative electrodes (connected to Au electrode on SiO_2). The sample was placed on a hot plate and the wires were bonded to the end of each Au electrode with conducting resin (H20E, EPO-TEK, Epoxy), followed by a heating at 110°C for 20 min to solidify the contact areas between the wires and the Au electrodes.[2] All fabrication processes in (a) (b) (c) were preformed in a 100-class modular cleanroom.

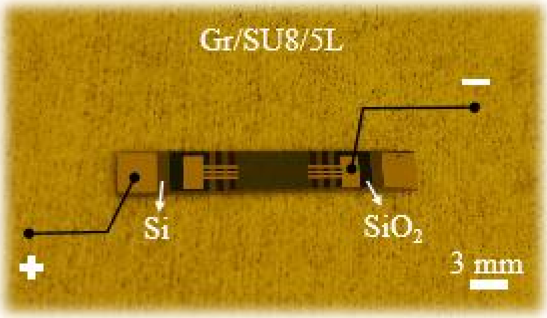

Figure S3. Photograph of the well-prepared Gr/SU8/5L sample with size of ~ 25 × 3 mm^2. Two independent wires are bonded at the end of the Au electrodes for bias voltage as schematically depicted.

2. Sample characterization

Sample characterization involves three parts: (a) Surface curvature and surface residue of the sample. (b) Characterization of SU8 spacers and nano pillars. (c) Raman spectra of graphene sheets.

(a) Surface curvature and surface residue of the sample

A relatively flat surface could be obtained near the center of the SiO_2/Si wafer.[2,3] As shown in Figure S4a, the natural bow of a SiO_2/Si substrate was measured by a laser interferometer (ZYGO OMP-035/M). The maximum surface curvature was ~ 7 nm within an area of 3 × 3 mm^2, which is comparable to the flatness reported elsewhere.[4,5] The relative heights along four scanning directions of the SiO_2/Si substrate are shown in Figure S4b, indicating an adequately flat surface compared to the gap distance. Hence, the samples were treated as a flat plate-to-plate model in our calculation.[2-5]

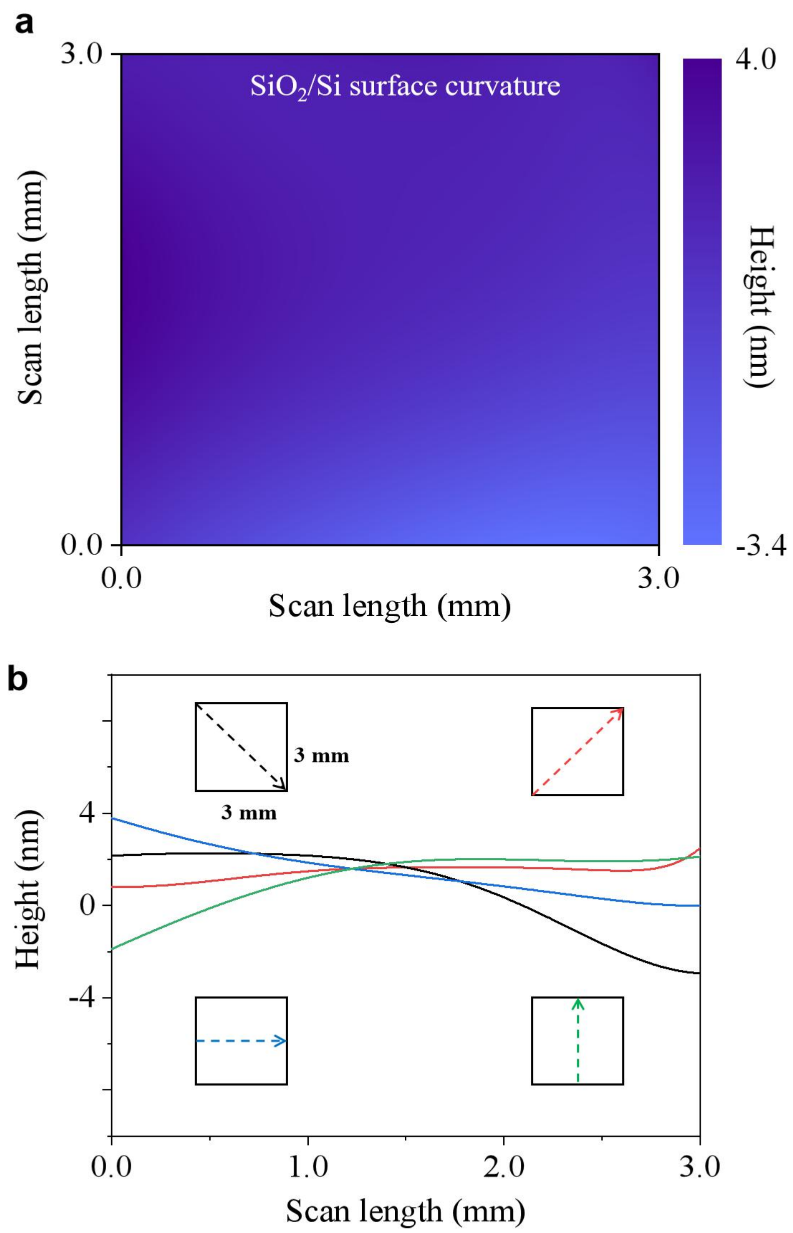

Figure S4. a) Surface curvature of the 3 × 3 mm^2 active region of a SiO_2/Si substrate, measured using the laser interferometer. b) Relative heights from four directions of the sample extracted from Figure S4a.

Paraffin is easier to remove from graphene than PMMA.[1] However, great efforts were still made to obtain a relatively clean graphene sheet as the thickness of the next SU8 spacer was only ~ 90 nm. After Step 10 in Section 1, the surface residues (mainly paraffin) of graphene sheet were characterized by AFM scanning as shown in Figure S5a. Graphene features a relatively flat surface with roughness less than ~ 1.5 nm. Some residues may have thickness of ~ 34 nm, ~ 40 nm, ~ 36 nm or ~ 55 nm (white-dashed circles) but they should not affect the

flatness and roughness of the next SU8 spacer. Figure S5b shows the 'topography' of Figure S5a where shading is accounted for which gives a more intuitive understanding of the height. Similar methods were employed in Figure S5d, Figure S5f, and Figure S5h.

We also characterized the surface roughness of SU8 spacer after spin coating onto the graphene sheet by AFM scanning. As illustrated in Figure S5c-S5d, the roughness of SU8 spacer is less than ~ 1 nm. Figure S5d shows that little particles surrounded by SU8 have thickness of ~ 10 nm (leveling to SU8 'substrate'), showing a good flatness of the SU8 spacer as expected. For comparison, AFM scanning of SU8 coated on a smooth SiO_2/Si was also presented, showing a similar roughness with that in Figure S5c. AFM scanning image of the first (top) graphene layer of a Gr/SU8/5L sample was shown in Figure S5g-S5h. The average roughness is ~ 1.5 nm with maximum thickness of the residues of ~ 54 nm. The areas of the residues are quite small compared with the scanning area of 8 × 8 μm^2. It is reasonable to assume that the heat conduction of the residues between the emitter and receiver is negligible during the NFTR experiments, which was also demonstrated by the good agreement of our computed and experimental data.

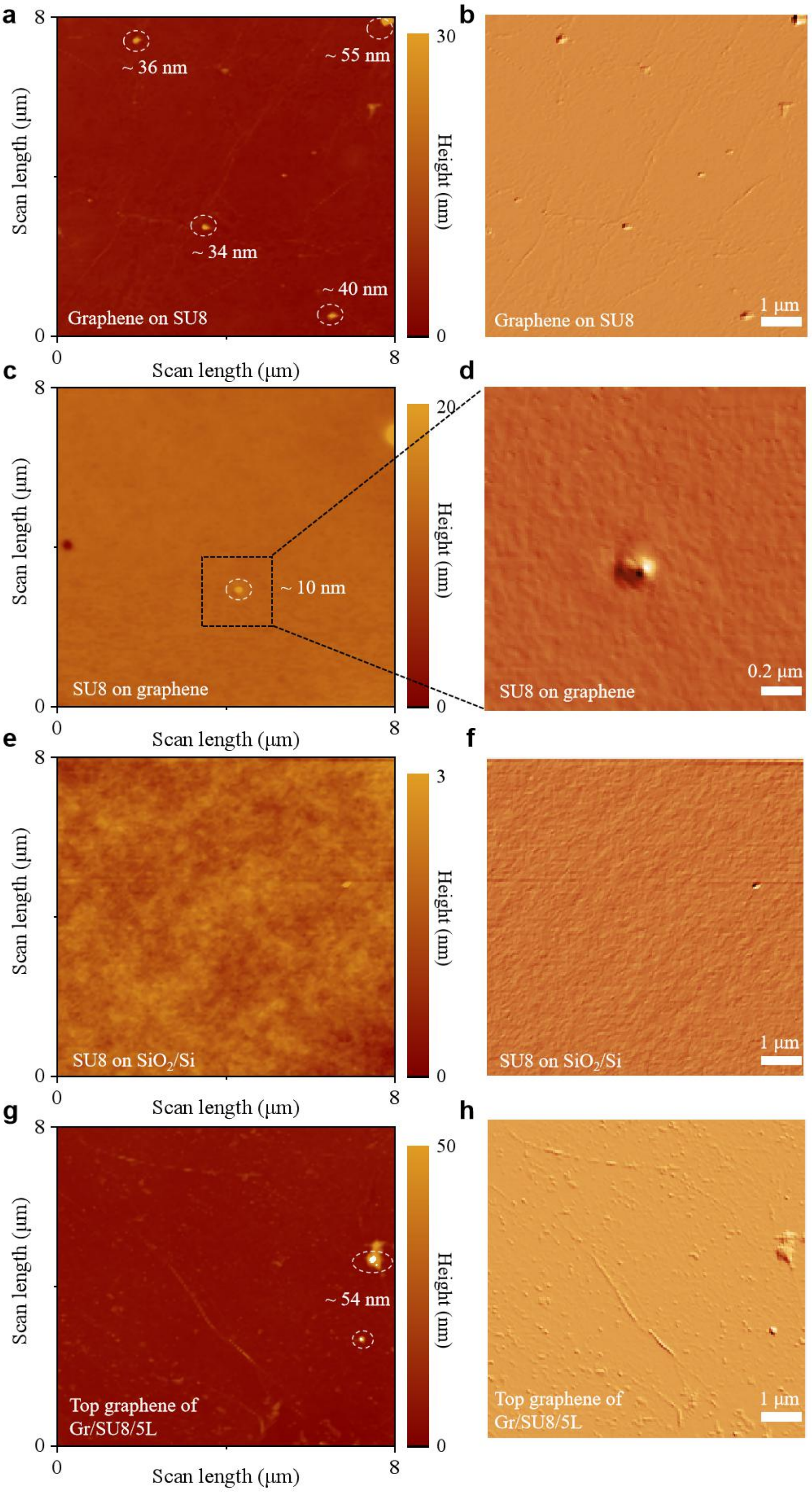

Figure S5. 8 × 8 μm^2 AFM scanning images of: a) Graphene on SU8. c) SU8 spacer after spin coating onto the graphene sheet. e) SU8 spin coated on a SiO_2/Si substrate. g) the top

graphene layer of our Gr/SU8/5L. (b), (d), (f) and (h) are the corresponding 'topography' maps with shading effects.

(b) Characterization of SU8 spacers and nano pillars

The thickness of the SU8 spacers (without graphene sheets) was confirmed by a film thickness measuring instrument (Filmetrics, F40-UV). As illustrated in Figure S6, twenty-five points were taken as samples within the active area (3 × 3 mm^2) for each of the layers. Data for each point was obtained by measuring the reflectance with wavelength from 400 nm to 1100 nm. The average standard deviation within such an area is less than 3 nm, indicating a quite flat surface could be obtained with the spin coating procedures. Figure S7 give one of the measurements in Figure S6 for the five layers of SU8 spacer, respectively, showing the agreement between the fitting and measured data. The thickness of each SU8 spacer could be confirmed as ~ 90 nm. Hence, considering the cleanliness of graphene sheets and the same experimental procedures, it was reasonable to assume that the thickness of each SU8 spacer of the Gr/SU8/5L sample was ~ 90 nm.

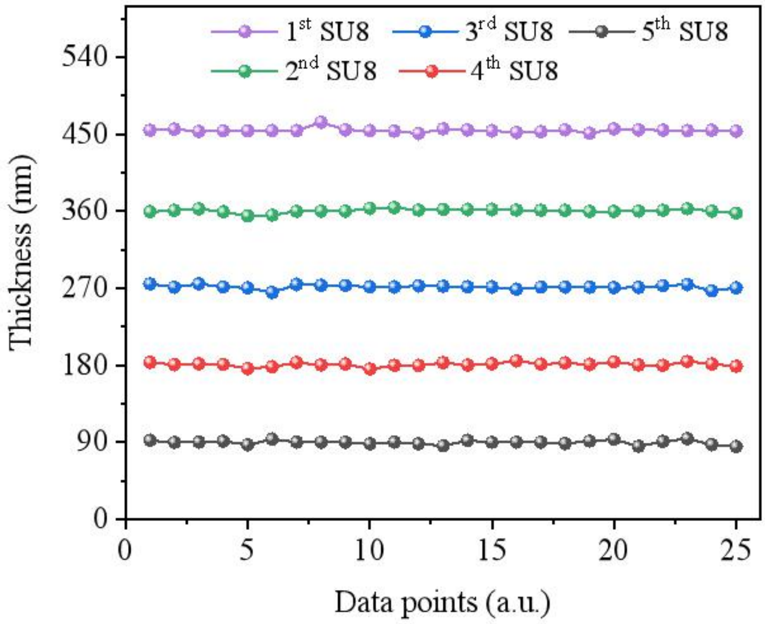

Figure S6. Thickness of the five layers of SU8 spacers within the 3 × 3 mm^2 active area measured at twenty-five data points.

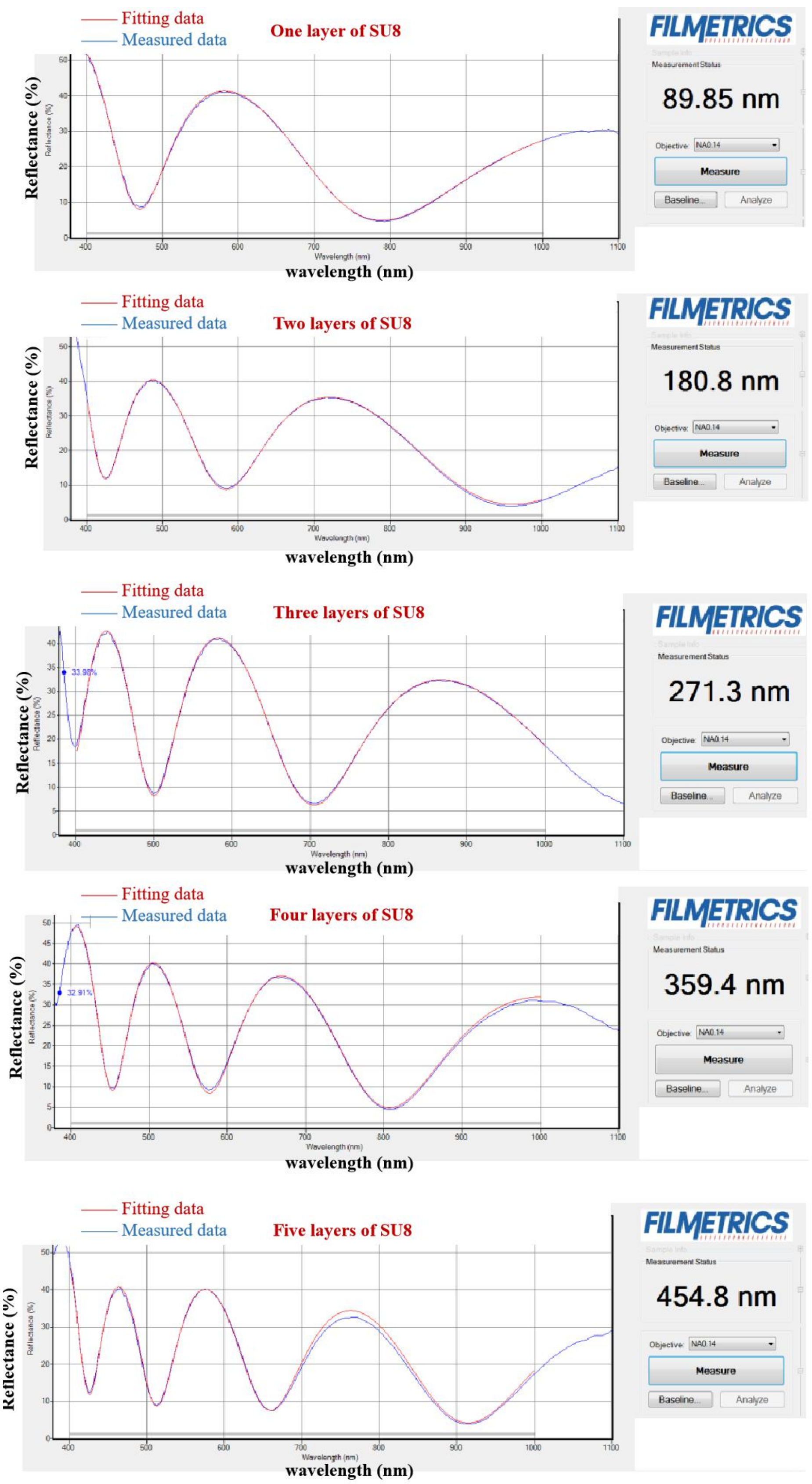

Figure S7. Thickness of each SU8 spacer obtained by the reflectance by Filmetrics (F40-UV). Red lines are the fitting data calculated by Cauchy model. Blue lines are the measured data.

The thicknesses of the SU8 nano pillars were equivalently confirmed by that of the SU8 film after UV treatments. Using the same experimental parameters in Step 22 in Section 1, the SU8 film was fabricated on SiO_2/Si substrate with thickness measured by Filmetrics (F40-UV) as well as an ellipsometer (not shown here). Twenty-five points for each sample were measured by Filmetrics within the active area. As shown in Figure S8, the maximum and minimum thickness of the SU8 films for different samples are ~ 63 nm and ~ 58 nm, respectively. The size of the top (first) SU8 spacer was 6 × 6 mm^2, which is larger than that of the active area and should offer an adequately flat 'substrate' for the nano pillars fabrication (as confirmed by Figure S5c and Figure S6). Hence, the thickness of the SU8 nano pillars was estimated within this range (i.e., ~ 58 nm to ~ 63 nm). Inset in Figure S8 is the optical image of a SU8 nano pillar with diameter of 20 μm. Note that during the NFTR experiment in our case, a total 95 g mass was applied to the emitter for mechanical stability which led to a small decrease of the thickness of the SU8 nano pillars (on the receiver), see SI Section 5 below.

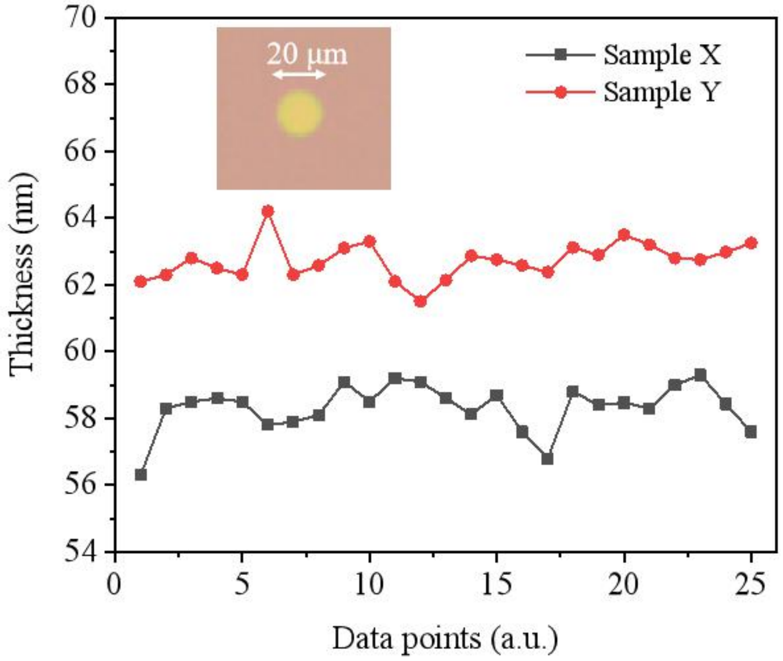

Figure S8. Thickness of the SU8 nano pillars are equivalently characterized by the SU8 films with same experimental parameters in Step 22 in Section 1. Sample X and sample Y give the minimum and maximum thicknesses. Inset shows the optical image of a SU8 nano pillar.

(c) Raman spectra of graphene sheets

Graphene sheets (of the Gr/SU8/5L) on different layers were in conductive contact through the Au/Ti electrodes and should have a similar Fermi level.[6] Hence, for simplicity, the Fermi level of the fifth graphene layer of a well-prepared Gr/SU8/5L sample was determined from the Raman spectra shown in Figure S9 without (Figure S9a) or with bias voltages (Figure

S9b). The higher intensity of the 2D peak (compared to the G peak) is indicative of single-layer graphene.[2,5,7,8] The Fermi level could be determined from the wavenumber w of the G peak according to the established equation: w_G - 1580 cm^{-1}=(42 cm^{-1}/eV) $|E_F|$.[8-10] For simplicity, the fifth graphene layer was then characterized by Raman spectra when applying different bias voltages, such as 5 V, 20 V, and 40 V. The measured G peaks of graphene with different bias voltages were shown in Figure S9b. The Fermi levels of the graphene sheets could be found as : ~ -0.3042 eV at 0 V, ~ -0.238 eV at 5 V, ~ -0.172 eV at 20 V, and ~ -0.127 eV at 40 V. w_G shifted to a smaller absolute value when more electron carriers flowed to the graphene sheets (with positive bias voltage), indicating a hole doping of the original graphene sheets. These measured values are only used for reference and the Fermi levels of the Gr/SU8/5L for the NFTR measurement in the main text were fitting parameters. The Fermi levels for different samples should feature a relatively small deviation due to the same sample fabrication procedures (i.e., samples came from a same batch).

The Fermi level for each sheet should be similar becuase they are in conductive contact through the gold electrode. The influence of fluctuations of the Fermi level on the NFTR was investigated to understand the sensitivity to sheets with mismatching Fermi levels. As shown in Figure S10, the NFTR between the Gr/SU8/5L heterostructures with Fermi level fluctuation versus different ΔT were plotted. The gap distance d is 54.7 nm and T_2 is 303.15 K. The five Fermi levels are set to:

d=54.7nm	Case A (main text)	Case B	Case C	Case D	Case E	Case F
E_{F1} (eV)	-0.11	-0.20	-0.11	-0.11	-0.13	-0.09
E_{F2} (eV)	-0.11	-0.17	-0.115	-0.105	-0.125	-0.095
E_{F3} (eV)	-0.11	-0.15	-0.12	-0.10	-0.12	-0.10
E_{F4} (eV)	-0.11	-0.13	-0.125	-0.095	-0.115	-0.105
E_{F5} (eV)	-0.11	-0.11	-0.13	-0.09	-0.11	-0.11

Table S1. List of the Fermi level fluctuations. Case A is assumed in the main text.

Firstly, with a larger fluctuation (e.g, Case B, the graphene Fermi level E_{F1} is almost 2-fold of E_{F5}), the error of the NFTR heat flux is ~ 24% compared to Case A, which does not match well with the experiment results. While with a smaller fluctuation, e.g., from Case C to Case F, the error of the NFTR heat flux compared to Case A is less than ~ 3.1%. Small or moderate local fluctuations do not meaningfully disrupt the NFTR. Hence, it is justified to assume a

Fermi level of -0.11 eV for each layer, which is also verified by the good agreement of the experiment data and theoretical calculation.

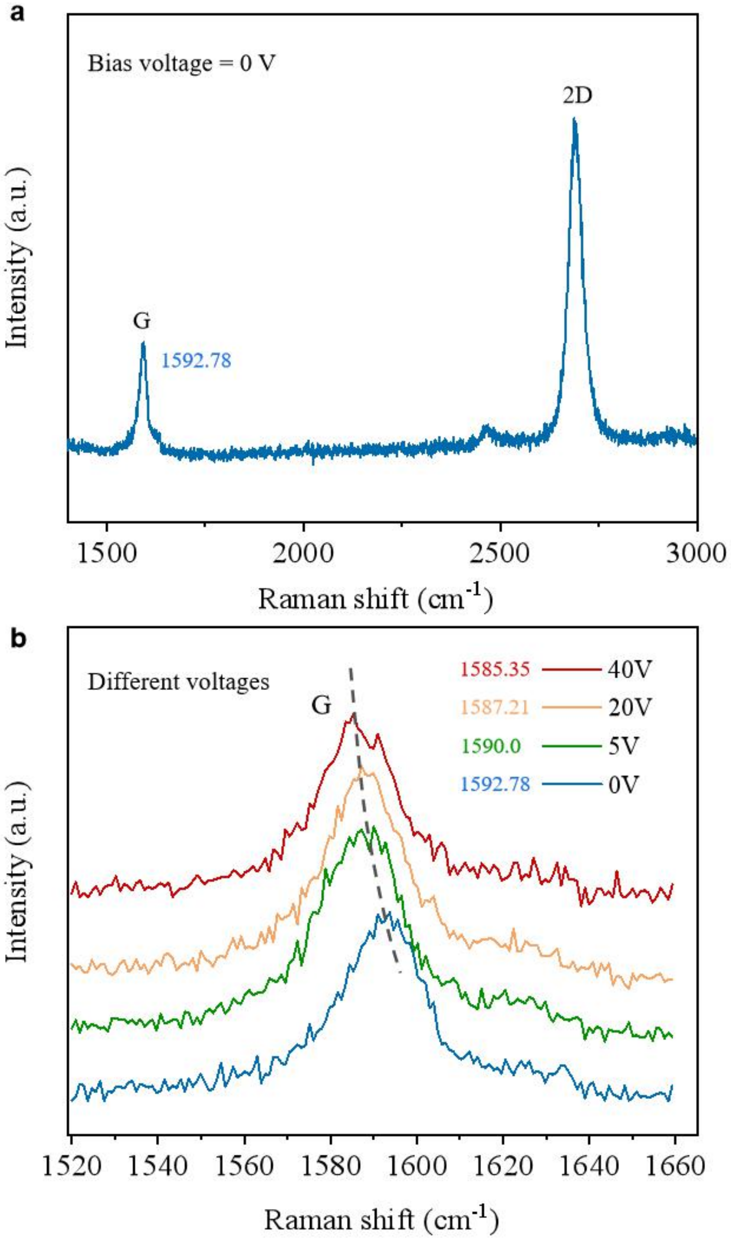

Figure S9. Raman spectra for the Gr/SU8/5L sample with different bias voltages. a) Raman spectra of the fifth graphene sheet was displayed without bias voltage (i.e., 0 V). b) Raman peaks from G resonance modes of the fifth graphene sheet were illustrated with bias voltages of 0 V, 5 V, 20 V, 40 V, respectively.

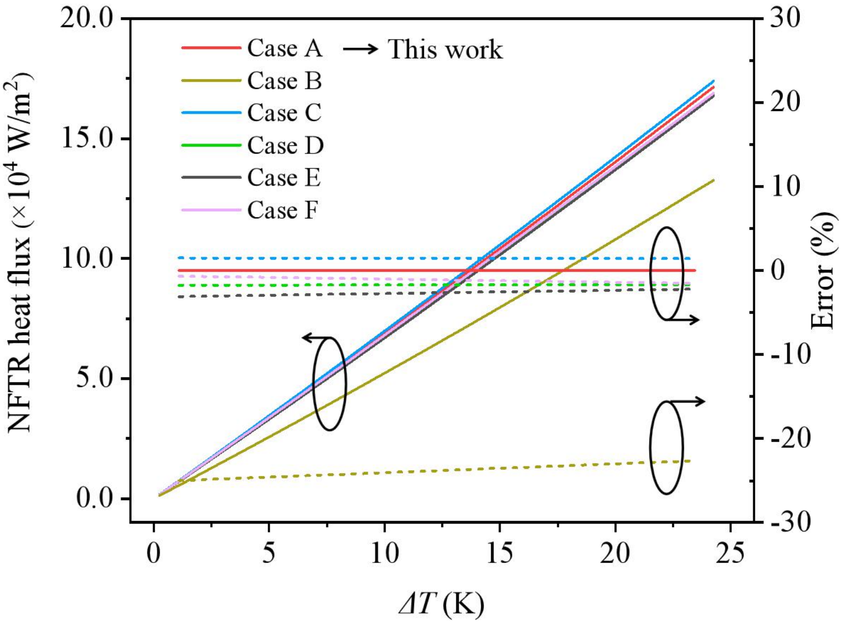

Figure S10. NFTR heat flux between the Gr/SU8/5L heterostructures with different amounts of Fermi level fluctuation. The gap distance d is 54.7 nm and T_2 is 303.15 K. Case A has the Fermi level of each graphene layer equal to -0.11 eV, which is used in the main text. Graphene Fermi level in other cases with a fluctuation around -0.11 eV are listed in Table R1.

3. Temperature calibration

The surface temperatures of the emitter and receiver were estimated by the relationship: T_2 (T_1) = T_{2b} (T_{1b}) ± P_{total} R_1. The measured value R_1 (unit: K/W) was the sum of the thermal resistances of the emitter (receiver), thermal conductive adhesive and the copper carrier (between the temperature detector and the thermal conductive adhesive). Hence R_1 could be obtained by:

$$R_1 = \frac{1}{2}\frac{(T_{1b} - T_{2b})}{P_{total}} \quad \text{(S1)}$$

As shown in Figure S11, two platinum resistance temperature detectors (thermistor, M222 class 1/3B, Heraeus, Germany) coated with a little thermal grease were buried into a hole beneath the top surface of the copper carriers to characterize the backside temperature T_{1b} of the emitter and T_{2b} of the receiver. In this case, the emitter and receiver were two identical SiO_2/Si samples with wires connected to the Au/Ti electrodes (not shown here), as the thermal resistance of the graphene-covered SU8 films could be ignored due to the huge width-depth ratio. The thin thermal grease was glued onto the interface between the emitter and receiver to minimize the contact thermal resistance, and a total 95 g mass (including copper carrier A) was added onto copper carrier A to strengthen the contact and mechanical stability of the

system[4,5] (not shown here). The measurement was done in a high vacuum environment (~2.8 × 10^{-5} Pa). R_1 from three independent measurements were shown in Figure S11, with range from ~ 7.49 K/W to ~ 11.5 K/W. Hence, R_1 was set at an average value of ~ 9.2 K/W during the NFTR experiments.

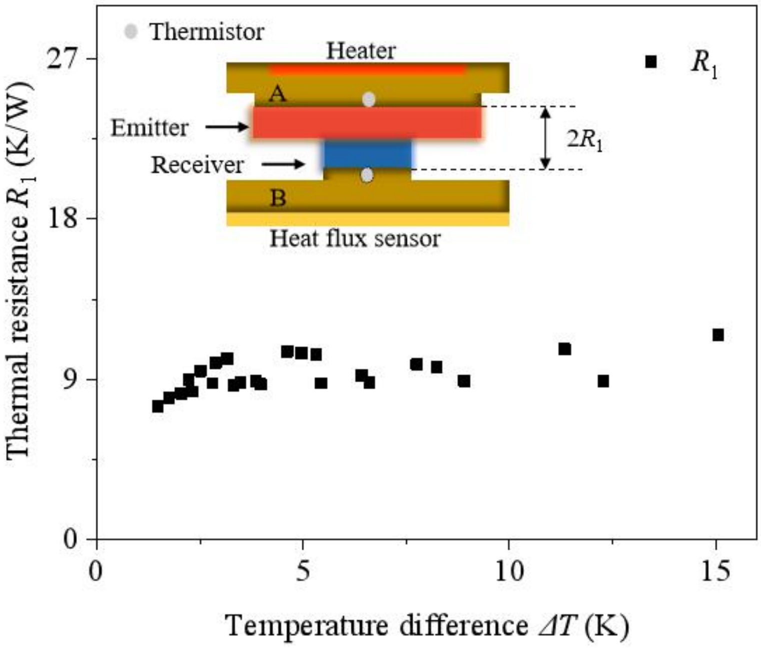

Figure S11. Measured value of R_1 with different ΔT. Inset shows the key experimental set-up. The sum thermal resistance of the emitter, thermal conductive adhesive and the copper carrier (between the temperature detector and the thermal conductive adhesive) was assumed to be same as that of the receiver.

4. Experimental system and measurement

Measurement of the NFTR was done in a vacuum chamber with a high vacuum environment (~2.8 × 10^{-5} Pa). The parallel alignment of the emitter and receiver was guaranteed by the SU8 nano pillars between them. Such a method for a fixed gap distance is widely employed.[4,5,11,12] Each point of the curves in Figure 3 in the main text was an average value from three possible active areas (i.e., 2.9 × 2.9 mm^2, 3 × 3 mm^2, and 3.1 × 3.1 mm^2) after four readings from the external heat flux meter (connected to the heat flux sensor below copper carrier B). Error bars were plotted due to the uncertainty of the active area. Temperature T_2 was maintained by a temperature electric controller (TEC) with an external source (Keithley, 2231A-30-3, Keithley instruments, USA) according to the thermal resistance R_1 and the total heat power P_{total}. Temperature T_{1b} and T_1 were then recorded after holding for at least 10 min to ensure thermal equilibrium.[2,5] Temperature T_{1b} and T_{2b} were obtained by reading the values of the platinum resistance temperature detectors via an external digital multimeter

(Keithley, DMM6500, Keithley instruments, USA). Figure S12 shows the whole experimental system. The emitter and receiver were controlled at the same bias voltages by external sources. The experimental data at other bias voltages with different ΔT were obtained with the same operations mentioned above. Before the NFTR experiment, some Gr/SU8/5L samples were used for Raman spectra measurement at different bias voltages. Since we found that under the bias voltage of ~ 40 V, the graphene Fermi level of our samples could reach ~ -0.1 eV to ~ -0.13 eV, which meets the needs of our theoretical prediction, we used the bias voltage only up to 40V.

The NFTR experiments with different bias voltages were challenging and highly dependent on the quality and cleanliness of the graphene sheets. To have a better chance of success of the NFTR experiments, one may employ polymer-free method,[13] or dry transfer method[14] for a better surface cleanliness, and may use a smaller size of the active area (e.g., 0.5 × 0.5 mm^2) to obtain a more flat surface. To fabricate such a small and complex system (intergrated with temperature measurement and Fermi level manipulation), one may refer to Ref. [15-19].

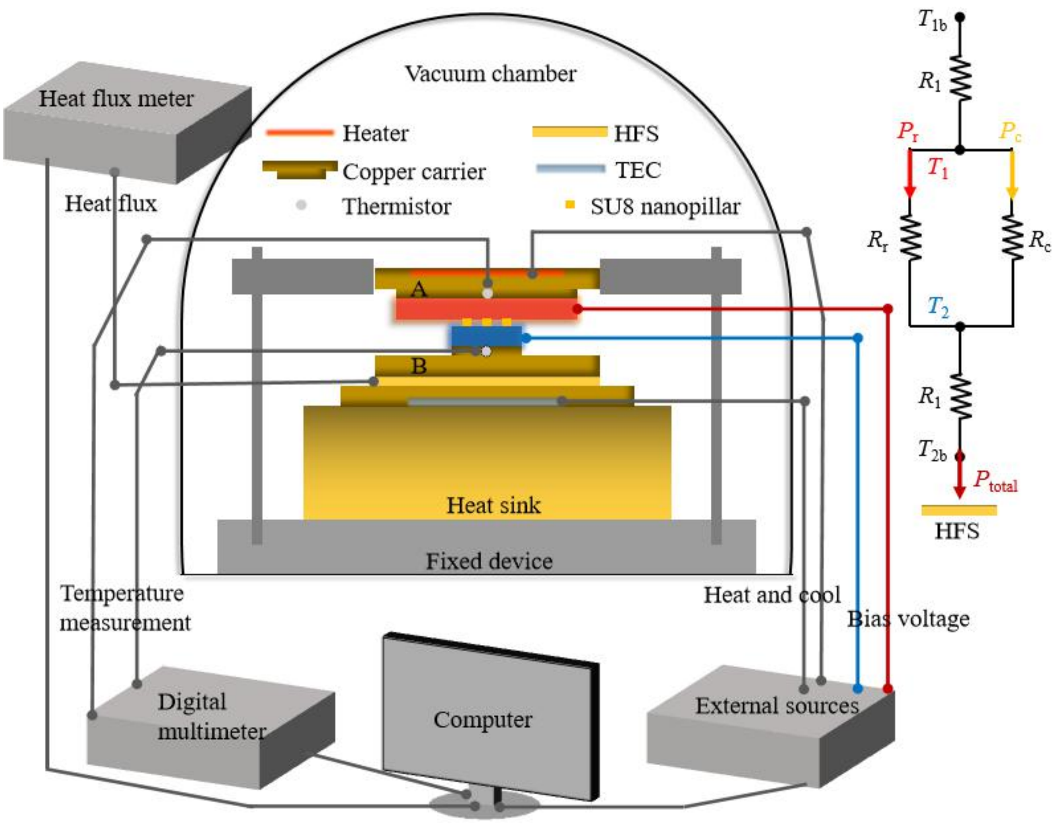

Figure S12. Schematic illustration of the whole measurement system. The equivalent thermal circuit was also displayed.

5. Gap distance and SU8 thermal conductivity

The gap distance between the emitter and the receiver was a calculated parameter according to the thickness and mechanical properties[4,20] of the SU8 nano pillars. As mentioned in the main text, a total 95 g mass was applied to the emitter for mechanical stability but led to a small decrease of the thickness of the SU8 nano pillars (as shown in Figure S13a). Young's modulus of SU8 is in the range of ~ 3.6 GPa to ~ 4.1 GPa at room temperature.[20] Hence, an average value of 3.85 GPa was considered in our case. A one-dimensional linear elastic analysis was employed according to Hooke's law. Displacement Δx could be calculated by:

$$\Delta x = t\frac{F_x}{E_{SU8}}, \tag{S2}$$

where t is the thickness of the SU8 nano pillar, F_x is the stress along the axial direction of the SU8 nano pillar, and E_{SU8} is the corresponding Young's modulus. When the 95 g mass was applied to the SU8 nano pillars with thickness in a range of ~ 58 nm to ~ 63 nm (SI Section 2), displacements of ~ 5.6 nm to ~ 6.1 nm would occur. Similar analyses (not shown here) could verify the results in Ref. [4] computed by Comsol Multiphysics. Thus, the gap distance between the emitter and receiver was within a range from ~ 52.4 nm to ~ 57 nm (i.e., d ~ 55 nm in the main text), which was also verified by the agreement between the computed and experimental data of the NFTR.

We also measured the NFTR between two identical ~ 305-nm-thick SiO_2/Si samples. SU8 nano pillars with thickness in a range of ~ 88 nm to ~ 92 nm (using the same experimental parameters as the SU8 spacer, see Step 7 in Section 1) were fabricated onto the receiver. According to elastic analysis, the gap distance between the emitter and receiver should be in a range of ~ 79.5 nm to ~ 83.1 nm. The computed and experimental data displayed in Figure S13b were consistent with this analysis. For comparison, heat flux within a gap distance range from 88 nm to 92 nm versus different ΔT were also calculated, showing a clear difference between the computed and experimental data. Temperature T_2 was maintained at 301.65 K ± 0.5 K. The dielectric functions of SiO_2 and Si were taken from Ref. [21].

The thermal conductivity has been verified by the NFTR experiment via a pair of 305-nm-thick SiO_2/Si sample (Figure S13b), as the experimental data of the NFTR (= measured total heat flux - heat conduction portion) agrees well with the prediction. In this case (305-nm-thick SiO_2/Si sample at a gap distance of 81.3 nm), the ratio of the heat conduction to the total heat flux is quite high (~ 58%; due to the large gap distance and consequently the near-field coupling is relatively small). We have obtained the heat conduction by subtracting the well-known NFTR of the 305-nm-thick SiO_2/Si sample[16,19] from the total heat flux, and plotted

the measured thermal conductivity (Figure S13c), which matches well with the value given by the SU8-3005 producer and Ref. [4]. The average value of the measured thermal conductivity is 0.207 ± 0.01 W m^{-1} K^{-1} (red-dotted line). Hence, the thermal conductivity of 0.2 W m^{-1} K^{-1} used in our work is reliable and reasonable.

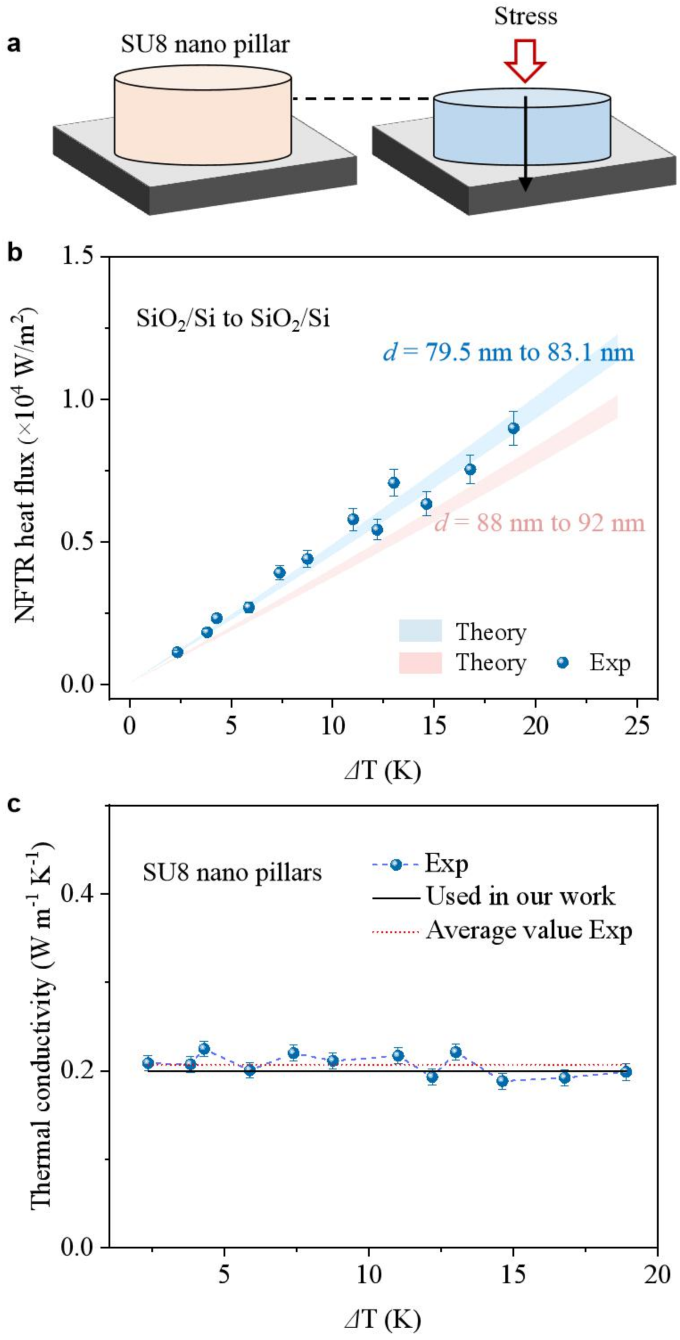

Figure S13. a) Schematic illustration of the condition of a SU8 nano pillar before (left) and after (right) adding the mass. b) Measured NFTR heat flux between two ~ 305-nm-thick SiO_2/Si samples with different ΔT within a gap distance range from 79.5 nm to 83.1 nm (i.e., d ~ 81.3 nm). The computed data for d = 79.5 nm to 83.1 nm (blue) and d = 88 nm to 92 nm (red) were given for comparison. Temperature T_2 was maintained at 301.65 K ± 0.5 K. c)

Verification of the thermal conductivity of the SU8 nano pillars. The red-dotted line is the average value of the measured thermal conductivity. Points are the experimental data of the thermal conductivity obtained from the heat conduction. The black-solid line represents the thermal conductivity of 0.2 W m^{-1} K^{-1} used in our work. Error bars were plotted due to the uncertainty of the active area (from 2.9 × 2.9 mm^2 to 3.1 × 3.1 mm^2).

6. Dielectric function of SU8 spacer

The dielectric function of SU8 (ε_{SU8}) was retrieved from the measured reflectance spectra using a Fourier transform infrared (FTIR) spectrometer. SU8 film was spin coated on a 95-nm-thick Au film (with 5-nm-thick Ti as adhesion layer on the Si substrate). The same UV lithography procedures in Step 8 in Section 1 were employed. SU8 films with thickness of 595 nm and 819 nm (which were both average values measured by the Filmetrics) were used for the reflectance spectra experiments with normal incidence for better signal noise ratio (SNR). The Multiple Lorentz-Drude oscillator model[22,23] given in the methods in the main text was employed for the dielectric function of SU8. Table S2 gives the parameters of the SU8 dielectric function. The error of the measured value and fitted value of each frequency point was put into a column vector, and the average value of the norm of the two column vectors (with data come from 595-nm and 819-nm SU8 thin film) was taken through MATLAB as the error function. The fitting and measured reflectance spectra in a wavelength range from 1.42 μm to 20 μm were illustrated in Figure S14a. The average relative error was less than 0.3% indicating a good agreement. The reflectance spectra within the color bands (covered a wavelength range from 9 μm to 20 μm) indicate negligible absorption (i.e., small loss) of the SU8 films. Figure S14b gives the real and imaginary portions of the dielectric function of SU8 described by the multiple Lorentz-Drude oscillator models.

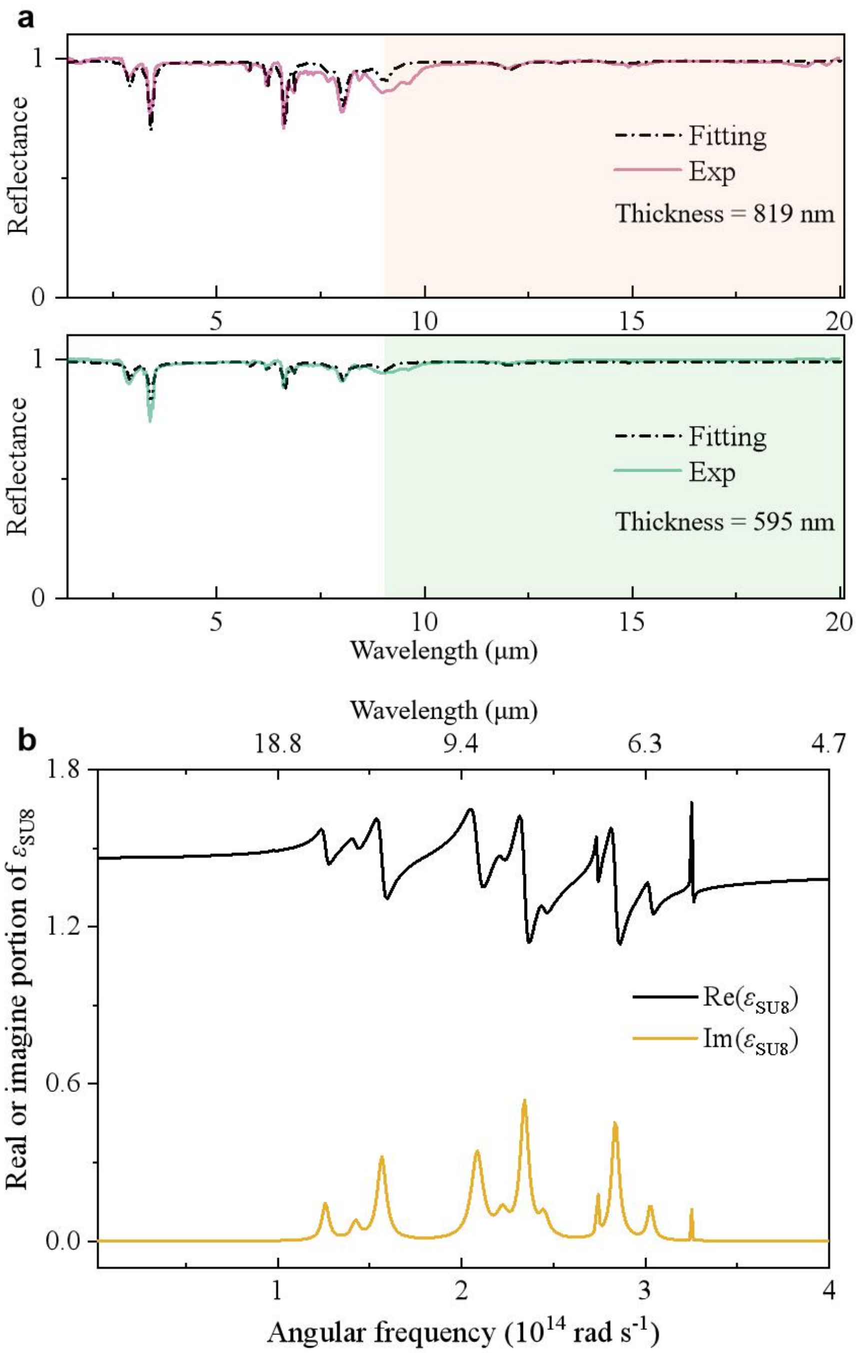

Figure S14. a) Measured (colored lines) and fitting data (dash-dotted lines) for the reflectance of the SU8 films with thickness of 819 nm and 595 nm, respectively. b) Real and imaginary parts of the dielectric function of the SU8 retrieved by the reflectance spectra.

ε_{inf} = 1.39999916	A_{1x} (a. u.)	A_{2x} (cm^{-1})	A_{3x} (a. u.)
x = 1	0.00146075124671989	3434.00449603104	0.0550829714785812
x = 2	0.00339324045214859	2929.00010065020	0.0342293909903620
x = 3	0.000254076611781735	1726.95250654918	0.000238835928456674
x = 4	0.00164505234500289	1607.32938462883	0.0123386061018492

x = 5	0.00766820735287619	1506.00012268947	0.0166791825465552
x = 6	0.000799976495587904	1454.55691091618	0.00408971338164520
x = 7	0.00199995712070057	1299.97538186534	0.0243073273473874
x = 8	0.0119999549628229	1243.54009888685	0.0230847195383060
x = 9	0.00299993349067481	1180.01066179183	0.0339948737217765
x = 10	0.0119999493588491	1107.00214537184	0.0362603031297629
x = 11	0.0116815750547869	830.973779283478	0.0366756925269940
x = 12	0.00242967416538911	755.762192815132	0.0378141204042466
x = 13	0.00499559061930623	668.504481832154	0.0353934972498788
x = 14	2.09166652558700e-05	520.673151122782	2.61640166279757e-05

Table S2. List of the parameters of the Lorentz-Drude model for the SU8 dielectric function.

7. Effect of heat conduction of SU8 nano pillars

The heat conduction of the SU8 nano pillars was calculated using Fourier's Law (here the temperature difference was taken as absolute value):

$$P_c = \frac{S\kappa\,|\,\Delta T\,|}{d}, \tag{S3}$$

where S is the sum area of eight SU8 nano pillars, κ is the thermal conductivity[4] (0.2 W m^{-1} K^{-1}), and d is the thickness of SU8 nano pillars which is set at an average value of ~ 54.7 nm (i.e., (52.4+57)/2 nm) in the calculations. Figure S15 show the ratios of the heat conduction P_c with respect to (a) the total heat power and (b) the NFTR power P_r at different ΔT for the Gr/SU8/5L samples at E_F = -0.11 eV, -0.17 eV, -0.25 eV, and -0.36 eV. For simplicity, the gap distance d was set at 54.7 nm, and temperature T_2 was 304.15 K. The ratio with respect to the total heat power reaches a value of ~0.13 at E_F = -0.11 eV and ~0.20 at E_F = -0.36 eV, indicating the contribution of the heat conduction to the total heat power is relatively small. Hence, in our case, the results of the NFTR power P_r (obtained by subtracting the calculated heat conduction P_c, whose thermal conductivity has also been verified by our experiment in a simple case, from the measured total heat power P_{total}) should be fairly close to the actual NFTR power P_r. To achieve a smaller heat conduction, one can reduce the area or increase the length of the SU8 nano pillars.[4]

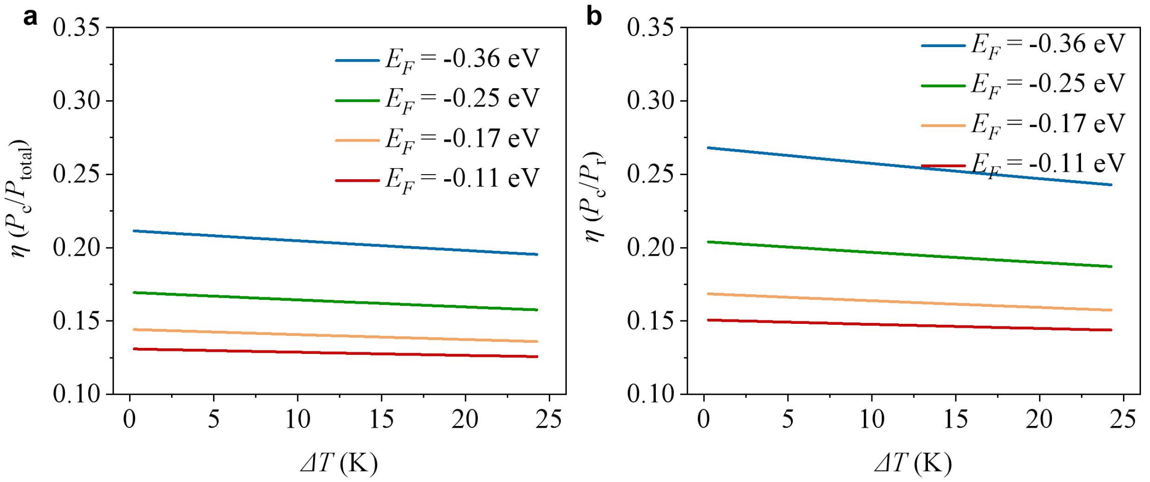

Figure S15. Ratio of the heat conduction P_c with respect to (a) the total heat power and (b) the NFTR power P_r at different ΔT for the Gr/SU8/5L samples with different graphene Fermi levels. Gap distance d was set at 54.7 nm, and temperature T_2 was 304.15 K.

8. Effect of number of layers of Gr/SU8 heterostructures for NFTR

We have theoretically investigated the effect of the number of layers of the Gr/SU8 heterostructures for NFTR. Calculations of the heat flux normalized with the corresponding BB limit at a fixed gap distance d = 50 nm were displayed in Figure S16a for Gr/SU8/1L, Gr/SU8/2L, Gr/SU8/3L, Gr/SU8/4L, and Gr/SU8/5L heterostructures with different E_F, respectively. Bulk SiO_2 was considered as a substrate. Gr/SU8 with infinite layers was also computed for comparison. Thickness t of each SU8 spacer was 90 nm, and ΔT was 10 K with T_2 set at 303.15 K.

As shown in Figure S16a, with the number of layers of Gr/SU8 increased, η is always increasing. Better enhancement could be observed with more Gr/SU8 layers and would eventually converge to a limit, due to more graphene SPP coupling modes. Points in black-dashed squares are the theoretical targets in this work. η of the Gr/SU8/5L heterostructures at E_F = -0.11 eV reaches ~ 1189, which is 1.21-fold larger than that of the Gr/SU8/1L heterostructures, and is ~ 98% of that of the Gr/SU8/infinite heterostructures. Without the contribution of the surface phonon polaritons (SPhPs) from the SiO_2 substrate, enhancement of the Gr/SU8/1L heterostructures could only reach ~ 77% of that of the Gr/SU8/infinite heterostructures. This enhancement difference is more pronounced with larger E_F. For example, when E_F = -0.36 eV, η of the five-layer structure is ~ 628, which is 1.51-fold of that of the single-layer structure (on the SiO_2/Si substrate) and 1.76-fold of that without SiO_2/Si substrate (not shown in the figure). With more Gr/SU8 layers, the contributions of the SPhPs from the substrate will be highly reduced due to the rapid attenuation of the evanescent

modes.[2] The photon tunneling probability ξ_p of each structure (on the SiO_2/Si substrate) at E_F = -0.36 eV were plotted in Figure S16b-S16g to show the SPP coupling modes. For the Gr/SU8/1L heterostructure, near-unity ξ_p enabled by coupled graphene SPPs from two interacting graphene sheets (of emitter and receiver) is restricted in two thin branches, which split at a lower β and merge at a larger β in a broad frequency range. ξ_p is near-unity in the region where the dispersion curves (not shown here) of the resonance modes overlap with the contour plot, demonstrating the important role of coupled SPPs in enhancing near-field heat transfer. When the number of layers of Gr/SU8 increases, more coupled SPPs from more graphene sheets are excited and enable near-unity ξ_p within more branches, e.g., four branches could be clearly observed in the Gr/SU8/2L heterostructures. Hence, in the Gr/SU8/5L heterostructures, ten branches with near-unity ξ_p enabled by the coupled SPPs of ten layers of graphene sheets form an almost continuous region in the *k*-space, accounting for the greater enhancement of NFTR than that of other graphene-covered SU8 heterostructures with fewer layers. For comparison, Gr/SU8/infinite heterostructures with continuous near-unity ξ_p (within the mid- and far-infrared frequency region) was also calculated.

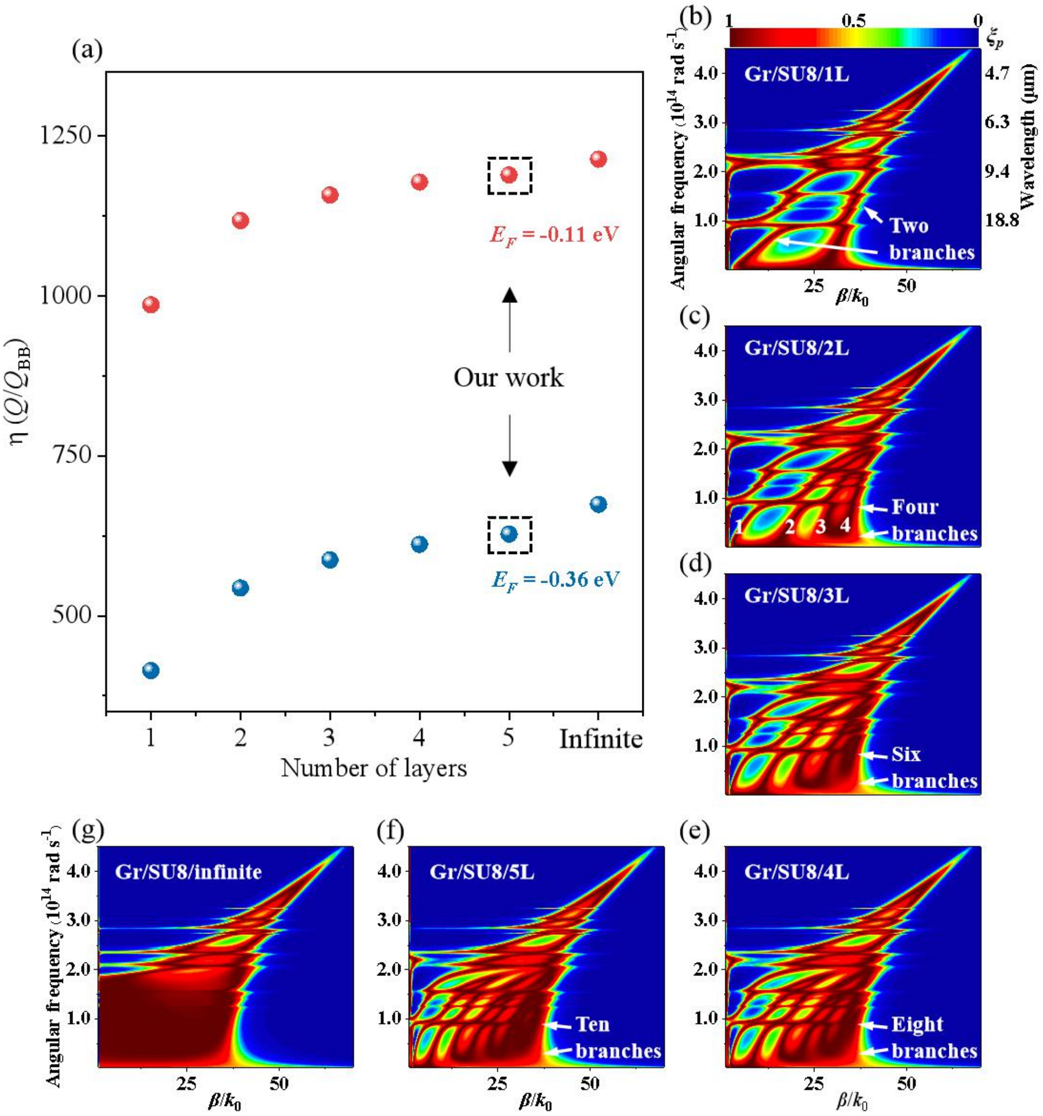

Figure S16. a) Enhancement normalized with the corresponding BB limit at a fixed gap distance d = 50 nm for Gr/SU8/1L, Gr/SU8/2L, Gr/SU8/3L, Gr/SU8/4L, Gr/SU8/5L, and Gr/SU8/infinite heterostructures at E_F = -0.11eV and -0.36 eV, respectively. b-g) show the corresponding contour plots of ξ_p.

References:

[1] W. S. Leong, H. Z. Wang, J. J. Yeo, F. J. Martin-Martinez, A. Zubair, P. C. Shen, Y. W. Mao, T. Palacios, M. J. Buehler, J. Y. Hong, J. Kong, *Nat. Commun.* **2019**, *10*, 867.

[2] K. Z. Shi, Y. C. Sun, Z. Y. Chen, N. He, F. L. Bao, J. Evans, S. L. He, *Nano Lett.* **2019**, *19*, 8082.

[3] M. Ghashami, H. Y. Geng, T. Kim, N. Iacopino, S. K. Cho, K. Park, *Phys. Rev. Lett.* **2018**, *120*, 175901.

[4] J. DeSutter, L. Tang, M. Francoeur, *Nat. Nanotechnol.* **2019**, *14*, 751.

[5] J. Yang, W. Du, Y. S. Su, Y. Fu, S. X Gong, S. L. He, Y. G. Ma, *Nat. Commun.* **2018**, *9*, 4033.

[6] N. H. Thomas, M. C. Sherrott, J. Broulliet, H. A. Atwater, A. J. Minnich, *Nano Lett.* **2019**, *19*, 3898.

[7] Z. Ni, Y. Wang, T. Yu, Z. Shen, *Nano Res.* **2008**, *1*, 273.

[8] H. G. Yan, F. N. Xia, W. J. Zhu, M. Freitag, C. Dimitrakopoulos, A. A. Bol, G. Tulevski, P. Avouris, *ACS Nano* **2011**, *5*, 9854.

[9] C. F. Chen, C. H. Park, B. W. Boudouris, J. Horng, B. S. Geng, C. Girit, A. Zettl, M. F. Crommie, R. A. Segalman, S. G. Louie, F. Wang, *Nature* **2011**, *471*, 617.

[10] J. Yan, Y. Zhang, P. Kim, A. Pinczuk, *Phys. Rev. Lett.* **2007**, *98*, 166802.

[11] M. P. Bernardi, D. Milovich, M. Francoeur, *Nat. Commun.* **2016**, *7*, 12900.

[12] W. Du, J. Yang, S. Zhang, N. Iqbal, Y. D. Dang, J. B. Xu, Y. G. Ma, *Nano Energy* **2020**, *78*, 105264.

[13] X. W. Zhang, C. Xu, Z. X. Zou, Z. H. Wu, S. Q. Yin, Z. L. Zhang, J. L. Liu, Y. Xia, C. T. Lin, P. Zhao, H. T. Wang, *Carbon* **2020**, *161*, 479.

[14] S. J. Yang, S. Choi, F. O. O. Ngome, C. J. Kim, S. Y. Choi, C. J. Kim, *Nano Lett.* **2019**, *19*, 3590.

[15] K. Kim, B. Song, V. Fernandez-Hurtado, W. Lee, W. H. Jeong, L. J. Cui, D. Thompson, J. Feist, M. T. H. Reid, F. J. Garcia-Vidal, J. C. Cuevas, E. Meyhofer, P. Reddy, *Nature* **2015**, *528*, 387.

[16] B. Song, D. Thompson, A. Fiorino, Y. Ganjeh, P. Reddy, E. Meyhofer, *Nat. Nanotechnol.* **2016**, *11*, 509.

[17] R. St-Gelais, L. Zhu, S. Fan, M. Lipson, *Nat. Nanotechnol.* **2016**, *11*, 515.

[18] A. Fiorino, D. Thompson, L. X. Zhu, B. Song, P. Reddy, E. Meyhofer, *Nano Lett.* **2018**, *18*, 3711.

[19] H. Salihoglu, W. Nam, L. Traverso, M. Segovia, P. K. Venuthurumilli, W. Liu, Y. Wei, W. J. Li, X. F. Xu, *Nano Lett.* **2020**, *20*, 6091.

[20] T. G. Xu, J. H. Yoo, S. Babu, S. Roy, J. B. Lee, H. B. Lu, *J Micromech. Microeng.* **2016**, *26*, 105001.

[21] E. D. Palik, *Handbook of Optical Constants of Solids*, Academic Press, San Diego, CA, **1985**.

[22] H. W. Verleur, *J Opt. Soc. Am.* **1968**, *58*, 1356.

[23] A. Ghanekar, M. Ricci, Y. Tian, O. Gregory, Y. Zheng, *Opt. Mat. Express* **2018**, 8, 2017.
